# Quantifying fecal and plasma short-chain fatty acids in healthy Thai individuals

**DOI:** 10.1016/j.csbj.2024.05.007

**Published:** 2024-05-08

**Authors:** Weerawan Manokasemsan, Narumol Jariyasopit, Patcha Poungsombat, Khwanta Kaewnarin, Kwanjeera Wanichthanarak, Alongkorn Kurilung, Kassaporn Duangkumpha, Suphitcha Limjiasahapong, Yotsawat Pomyen, Roongruedee Chaiteerakij, Rossarin Tansawat, Chatchawan Srisawat, Yongyut Sirivatanauksorn, Vorapan Sirivatanauksorn, Sakda Khoomrung

**Affiliations:** a Department of Biochemistry, Faculty of Medicine Siriraj Hospital, Mahidol University, Bangkok, Thailand; b Siriraj Center of Research Excellent in Metabolomics and Systems Biology (SiCORE-MSB), Faculty of Medicine Siriraj Hospital, Mahidol University, Bangkok, Thailand; c Siriraj Metabolomics and Phenomics Center, Faculty of Medicine Siriraj Hospital, Mahidol University, Bangkok, Thailand; d Thailand Metabolomics Society, Bangkok, Thailand; e SingHealth Duke-NUS Institute of Biodiversity Medicine, National Cancer Centre Singapore, Singapore; f Translational Research Unit, Chulabhorn Research Institute, Bangkok, Thailand; g Center of Excellence for Innovation and Endoscopy in Gastrointestinal Oncology, Division of Gastroenterology, Department of Medicine, Faculty of Medicine Chulalongkorn University, Chulalongkorn University, Bangkok, Thailand; h Department of Food and Pharmaceutical Chemistry, Faculty of Pharmaceutical Sciences, Chulalongkorn University, Bangkok, Thailand; i Metabolomics for Life Sciences Research Unit, Chulalongkorn University, Chulalongkorn University, Bangkok, Thailand; j Department of Surgery, Faculty of Medicine Siriraj Hospital, Mahidol University, Bangkok, Thailand; k Center of Excellence for Innovation in Chemistry (PERCH-CIC), Faculty of Science, Mahidol University, Bangkok, Thailand

**Keywords:** Gut microbial metabolite, Quantitative analysis, Gas chromatography-mass spectrometry, Short-Chain Fatty Acids, Feces, Plasma

## Abstract

Short-chain fatty acids (SCFAs) are involved in important physiological processes such as gut health and immune response, and changes in SCFA levels can be indicative of disease. Despite the importance of SCFAs in human health and disease, reference values for fecal and plasma SCFA concentrations in healthy individuals are scarce. To address this gap in current knowledge, we developed a simple and reliable derivatization-free GC-TOFMS method for quantifying fecal and plasma SCFAs in healthy individuals. We targeted six linear- and seven branched-SCFAs, obtaining method recoveries of 73–88% and 83–134% in fecal and plasma matrices, respectively. The developed methods are simpler, faster, and more sensitive than previously published methods and are well suited for large-scale studies. Analysis of samples from 157 medically confirmed healthy individuals showed that the total SCFAs in the feces and plasma were 34.1 ± 15.3 µmol/g and 60.0 ± 45.9 µM, respectively. In fecal samples, acetic acid (Ace), propionic acid (Pro), and butanoic acid (But) were all significant, collectively accounting for 89% of the total SCFAs, whereas the only major SCFA in plasma samples was Ace, constituting of 93% of the total plasma SCFAs. There were no statistically significant differences in the total fecal and plasma SCFA concentrations between sexes or among age groups. The data revealed, however, a positive correlation for several nutrients, such as carbohydrate, fat, iron from vegetables, and water, to most of the targeted SCFAs. This is the first large-scale study to report SCFA reference intervals in the plasma and feces of healthy individuals, and thereby delivers valuable data for microbiome, metabolomics, and biomarker research.

## Highlights

• Fast and accurate derivatization-free GC-TOFMS method to quantify fecal and plasma SCFAs.
• The absolute concentrations of fecal and plasma SCFAs from 157 healthy individuals were quantified.
• The concentrations of SCFAs in healthy individuals were found to be independent of age and gender.

## Graphical Abstract

**Figure:**
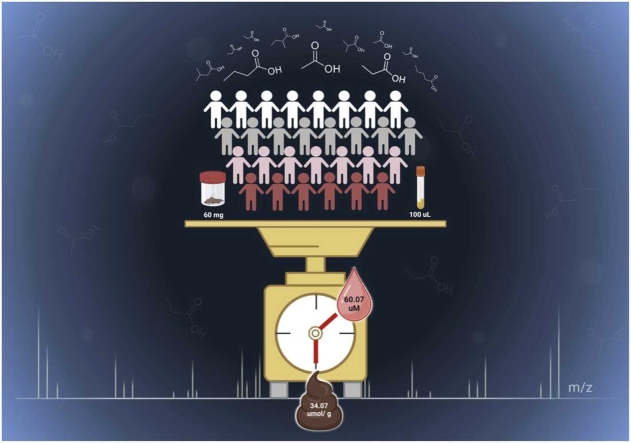

## 1 Introduction

The gut microbiome plays an important role in human health and disease [1], [2]. Changes in the gut microbiome and its metabolic byproducts have been shown to reflect health status [3], [4], [5]. Bacteroidetes and Firmicutes are known to produce short-chain fatty acids (SCFAs) through the fermentation of non-digestible dietary fibers [6], [7], [8], [9]. SCFAs are key energy sources for intestinal epithelial cells and are involved in several cellular processes, *e.g.,* chemotaxis, cell proliferation, cell differentiation, and gene expression [10], [11]. These key bacterial metabolites not only indicate human health status, but also influence the diverse functions of various physiological processes related to health and disease [12], [13]. For instance, decreased levels of SCFAs or SCFA-producing bacteria have been associated with the development of diseases such as Parkinson’s disease [14], irritable bowel syndrome [15], type 1 diabetes [16], and Alzheimer’s disease [17]. Although SCFA levels appear to be crucial in many clinical applications, only a few studies have reported quantitative data on healthy people, including those in the United Kingdom (N = 3), Belgium (N = 12), and Malaysia (N = 50) [18], [19], [20]. Those studies reported that the most common SCFAs were Ace, Pro, and But, accounting for approximately 90% of all fecal SCFAs. Previous studies reported the three major SCFAs, but the minor SCFAs, such as the branched SCFAs were not reported. Furthermore, it is crucial to acknowledge that the levels of SCFAs might differ greatly among individuals owing to various factors, especially among ethnic populations. Currently, there are few available data for comparing SCFAs among individuals [21].

Gas chromatography-mass spectrometry (GC-MS) is an analytical technique that is widely used to analyze small molecules, especially volatile and semi-volatile compounds. The identification and quantification of SCFAs by GC-MS often requires chemical derivatization prior to GC-MS analysis. Although derivatization is a well-established method, it is a multistep, time-consuming procedure, making it less suitable for cohort studies [22], [23], [24]. To overcome these issues, a derivatization-free method for the analysis of SCFAs using GC-MS has been proposed [25]. However, the early studies showed low recoveries of various SCFAs, *e.g.,* heptanoic acid (Hep; 45.5%−51.3%, N = 5), hexanoic acid (Hex; 61.1%−85.0%, N = 5) [26], and Ace (65%−74%, N = 3) [27]. Recently, Han et al. reported a derivatization-free GC-MS method for analyzing seven SCFAs in mice feces with excellent recoveries ranging from 89%− 105% [25]; the method, however, suffers from a long sample-preparation time of 3.5 h per sample.

There has been no report of a fast, rapid, and reliable method for quantifying fecal and plasma SCFAs in human samples suitable for large-scale investigations that includes comprehensive method validation. Therefore, our objectives were: (I) to develop a simple, fast, and reliable method for quantitative analysis of fecal and plasma SCFAs in a large cohort study; and (II) quantify fecal and plasma SCFAs from a medically confirmed healthy population (N = 157) and investigate the correlations of SCFAs with dietary nutrients, age, and sex.

## 2 Experimental procedures

### 2.1 Participant details and sample collection

Samples were collected at King Chulalongkorn Memorial Hospital. This study was approved by the Institutional Review Board of the Faculty of Medicine, Chulalongkorn University, Bangkok, Thailand (No. 057/62 and No. 372/64). The human studies reported here abide by the Declaration of Helsinki principles. Fecal and blood plasma samples were collected from 157 healthy volunteers aged 18–60 years (N = 60 from cohort 2019 and N = 97 from cohort 2022). Blood chemistry, stool features, and stool frequency were recorded and analyzed. Dietary details (*e.g.*, meals, ingredients, and amounts) were recorded for three consecutive days. Dietary intake included the estimated amount of ingredients in tablespoons or teaspoons. These data were converted to amounts of nutrients (*e.g.*, g or mg of carbohydrates, proteins, fats, and vitamins) and energy intake (kcal). Dietary records were analyzed using the Mahidol Inmucal program. All volunteers had normal renal function and blood pressure, no underlying diseases, and were not taking any medication. All the healthy volunteers were medically confirmed using the generally accepted normal ranges for blood chemistry parameters (*e.g.*, fasting blood glucose, cholesterol, triglyceride), and disease-indicated biomarkers (*e.g.*, AST, ALT, creatinine, and eGFR). In addition, chest X-ray results were evaluated by clinicians.

### 2.2 Chemicals, reagents, and standards

Analytical-grade hydrochloric acid (HCl) fuming 37%, diethyl ether (DE), and anhydrous acetic acid were purchased from Merck (Darmstadt, Germany). Analytical-grade anhydrous sodium sulfate, butyric acid, propionic acid, 2-methylpropionic acid, pentanoic acid, 2-methylbutanoic acid, 3-methylbutanoic acid, 3-methylpentanoic acid, 4-methylpentanoic acid, hexanoic acid, 2-methylhexanoic acid, 4-methylhexanoic acid, and heptanoic acid were purchased from Sigma-Aldrich (MO, USA). Propionic-d_5_ acid (Pro-D_5_), which was used as the internal standard (IS), was purchased from CDN Isotope Inc. (Quebec, Canada). Milli-Q water was provided by Milli-Q Advantage A10 Water Purification System from Merck (Darmstadt, Germany) with resistivity value of 18.2 MΩ·cm at 25 °C; ≤ 5 ppb.

### 2.3 Fecal and plasma sample collection and extraction

After collection, fecal samples were immediately homogenized and aliquoted before being stored at − 80 °C until use. Pooled fecal samples were prepared by mixing ∼0.5 g of all 60 (cohort 2019) and 97 (cohort 2022) fecal samples. To extract SCFAs from fecal samples, 60 mg of a fecal sample was extracted with 800 µL of Milli-Q water, 300 µL of 5 M HCl, and 1 mL of DE containing 20 µg of (Pro-D_5_). This process was prepared on ice because of the low boiling point of DE. The sample mixture was centrifuged at 5200 × *g* at − 10 °C for 15 min. After centrifugation, the supernatant (DE fraction) was transferred to a fresh conical tube containing 400 mg of Na_2_SO_4_. The sample residual was re-extracted with 1 mL of DE, and two extracts were combined before centrifugation at 5200 × *g* at − 10 °C for another 15 min. After centrifugation, 1 mL of the supernatant was transferred to a vial for GC-TOFMS analysis.

**Fig. 1:**
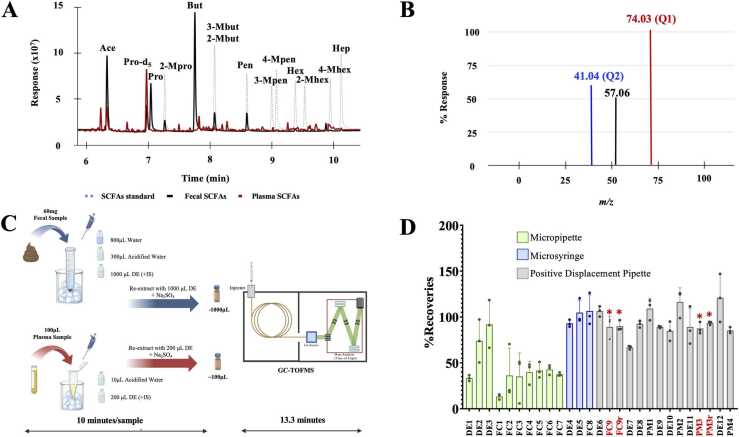
Development of the SCFA extraction protocol for fecal and plasma samples. (A) Overlaid GC-MS chromatograms of SCFAs in the standard solution, pooled fecal samples, and pooled plasma samples. (B) The mass spectrum showing the quantifier mass and qualifier mass. (C) Overview of SCFA extraction and analysis procedure. (D) Recoveries from SCFA extraction optimization (* denotes the final optimized conditions that were used to extract fecal SCFAs and plasma SCFAs). Abbreviations: DE, an extraction protocol that used diethyl ether solvent; FC, an extraction protocol using pooled fecal samples; PM, an extraction protocol using plasma sample; FC9r and PM3r, repeated conditions of FC9 and PM3, respectively. The different conditions are indicated in more detail in Supplementary table S2.

After the collection, blood samples were centrifuged at 2500 × *g* at 4 °C for 15 min [28], [29]. After centrifugation, the plasma layer was collected and aliquoted before being stored at − 80 °C until use. A pooled plasma sample was prepared by mixing 100 µL of all 60 (cohort 2019) and 97 (cohort 2022) plasma samples. To extract SCFAs, 100 µL of a plasma sample was extracted with 10 µL of 5 M HCl and 200 µL of DE containing 0.5 µg of propionic-d_5_ acid. The sample mixture was centrifuged at 5200 × *g* at − 10 °C for 15 min. After centrifugation, the supernatant (DE fraction) was transferred to a fresh Eppendorf tube containing 30 mg of Na_2_SO_4_. The sample residual was re-extracted with 200 µL of DE, and the two extracts were combined before being centrifuged at 5200 × *g* at − 10 °C for another 15 min. Finally, 100 µL of supernatant was transferred to a GC vial for GC-MS analysis. An overview of the steps of sample preparation and GC-TOFMS analysis is given in Fig. 1.

### 2.4 Gas chromatography-mass spectrometry analysis

The analysis was performed using a LECO Pegasus BT GC-TOFMS (Leco Corporation, St. Joseph, MI, USA). Chromatographic separation was performed using a DuraBond-FFAP column (length, 30 m × internal diameter, 0.25 mm i.d. × film thickness, 0.25 µm; Agilent Technologies, USA). Inlet temperature was 200 °C. Helium was used as a carrier gas at a flow rate of 1 mL/min. The injection volume was 1 µL. A split ratio of 60:1 was used for fecal samples, whereas plasma samples were analyzed in splitless mode. The initial oven temperature was 40 °C and increased to 240 °C at 15 °C/min. The transfer line temperature was 250 °C. TOFMS was operated in positive mode using an electron ionization (EI) ion source, with an ionization energy of 70 eV at 230 °C. The solvent delay was 300 s. MS was operated in full-scan mode, collecting *m/z* data ranging from 30 to 400 at a data acquisition rate of 20 spectra/s.

### 2.5 Limit of detection (LOD) and limit of quantification (LOQ)

Seven and eleven calibration solutions of 13 SCFA standards and an IS were prepared for fecal and plasma analyses, respectively. The calibration solutions were analyzed in triplicate before constructing the calibration curves, which ranged from 0.05 to 50 ng/µL and 0.003 to 70 ng/µL for fecal and plasma analysis, respectively. The correlation coefficients (R^2^) for all the SCFA calibration curves were greater than 0.99. The LOD and LOQ were calculated as follows on the basis of a previous publication [30]: LOD = 3 × SD/m and LOQ = 10 × SD/m, where SD is the standard deviation and m is the slope of the calibration curve.

### 2.6 Intraday and interday experiments

Intraday and interday experiments were performed to determine the precision and reproducibility of the methods. The mixed SCFA standard solutions were prepared at 20 ng/µL and 5 ng/µL for fecal and plasma analyses, respectively. The intraday experiment was conducted with 10 replicates within one day, whereas the interday experiment was conducted for three consecutive days, with 10 replicates each day. The mean and %relative standard deviation (%RSD) values were calculated for the retention time and peak area. We chose < 20% as an acceptable limit for the %RSD.

### 2.7 Matrix effect

The matrix effect in the fecal or plasma samples was evaluated by spiking individual SCFA standards at final concentrations of 20 ng/µL (fecal samples) or 5 ng/µL (plasma), followed by the sample preparation previously described. SCFA calibration curves in the matrix extract and in the DE solutions were constructed and compared (Table 1). The matrix effect was determined by comparing the slope of the matrix-matched calibration (in the matrix extract) and the normal calibration (in DE solvent). The percentage matrix effect was determined as follows:

$$\%\;\mathrm{Matrix}\;\;\mathrm{effect}\;\;\left(\mathrm{ME}\%\right)=\frac{(\mathrm{slope}\;\;\mathrm{of}\;\;\mathrm{matrix}-\mathrm{matched}\;\;c\mathrm{alibration})\times 100\%}{\left(\mathrm{slope}\;\;\mathrm{of}\;\;\mathrm{normal}\;\;\mathrm{calibration}\right)}$$

**Table 1:** Summary of GC-TOF-MS method validation.

Method Validation	Parameters	Fecal sample conditions	Plasma sample conditions
Matrix effect (%)^a^	The gradient of calibration curve from extracted matrix solvent compared with DE solvent	429 − 600	118 − 143
Recovery (%)^a^	Spiked mix standards before extraction	73 − 88	93 − 134
Sensitivity^b^	LOD	5 − 50 ng/mL	0.007 − 5.14 µM
	LOQ	14 − 152 ng/mL	0.024 − 17.133 µM
Precision (%RSD)^b^	Peak Area, Intraday (N = 10)	1 − 3	0 − 6
	Peak Area, Interday (N = 3)	6 − 11	2 − 17
	RT, Intraday (N = 10)	0.00 − 0.28	0.00 − 0.29
	RT, Interday (N = 3)	0.00 − 0.01	0.00 − 0.47

DE, diethyl ether; LOD, limit of detection; LOQ, limit of quantification; RSD, relative standard deviation; RT, retention time;

a Refers to full detail in Table S3

b Refers to full detail in Table S4

The percent matrix effect was calculated and shown in Table S3.

### 2.8 Recovery study

Pooled fecal and plasma extracts were spiked with mixed SCFA standards at final concentrations of 20 ng/µL (fecal samples) and 5 ng/µL (plasma samples), followed by the above- mentioned sample preparation. The measured analyte concentrations were used to determine the recovery after the extraction process. The recoveries were calculated using the following formula:

$$\%\;\mathrm{Recovery}=\frac{\mathrm{Measured}\;\;\mathrm{concentration}\;\;\mathrm{after}\;\;e\mathrm{xtraction}\times 100\%}{\mathrm{Theoretical}\;\;\mathrm{concentration}}$$

### 2.9 Metabolite identification, data processing, and data analysis

The GC-MS data were processed using ChromaTOF software (version 5.51.06.0.64572; LECO Corporation, St. Joseph, MI, USA). Metabolite identification was conducted using the standard protocol (Level 1), by comparing the mass spectrum profiles and retention times of unknown metabolites with those of reference standards [31]. The quantitative analysis utilized the matrix-matched calibration curve [32]. Randomization was employed in the analysis to exclude batch effects and other non-biological factors.

The SCFAs were excluded from the analysis if they were present in less than 80% of the population. The missing values of SCFAs were imputed by mean, and further used for univariate and multivariate analysis following our previous publication [33]. The comparison of SCFAs among other factors (*e.g.*, age or sex) were assessed using Wilcoxon Signed-Rank test adjusted by.

FDR-adjustment method (Benjamini-Hochberg) (p-value < 0.05) using R (version 4.3.3). The absolute weight of each nutrient, caloric distribution, and comparable percentage of dietary reference intake were analyzed using the Mahidol Inmucal program [34]. Correlations of fecal and plasma SCFAs with micronutrition, age, gender, BMI, fasting blood glucose, cholesterol, and triglyceride levels were assessed using Spearman’s correlation without data imputation. Differences were considered statistically significant when the FDR rate-adjusted p-value was < 0.05.

## 3 Results

### 3.1 Implementation and validation of derivatization-free GC-TOFMS methods

We used a DB-FFAP column to separate six linear and seven branched SCFAs, using Pro-D5 as the IS within 13.3 min (Fig. 1A). We characterized reference standards to determine their retention times and mass spectrum profiles for identification and quantification of SCFAs in real samples. Based on a previous publication [35], we designated the most intense ion of each SCFA as the quantifier ion (Q1), and the second-most intense ion as the qualifier ion (Q2). The ion selection criteria were: I) they had to be relatively high in intensity, II) they had to be unique ions at a specific retention time, III) they had to be stable ions, and IV) they had to have a similar fragment ratio to that detected in reference standards (Table S1). For instance, the mass spectrum of 2-methylbutanoic acid (2-Mbut) produced three fragments with *m/z* values of 41.04, 57.06, and 74.03 (Fig. 1B). The most intense fragment ion (*m/z* 74.03) was chosen as Q1, and the second-most intense fragment ion (*m/z* 41.04) was chosen as Q2. The ratio of the ion intensities of Q1 and Q2 was used to confirm the presence of 2-Mbut. Table S1 provides a list of Q1 and Q2 ions for all the targeted SCFAs. This procedure enhanced the accuracy of SCFA measurements in the presence of co-eluting peaks. For example, 3-Mbut (Q1 = *m/z* 60.02) and 2-Mbut (Q1 = *m/z* 74.03) co-eluted at 8.13 min (Fig. 1A, Table S1).

Owing to the different concentrations of SCFAs in feces and plasma, sample preparation protocols were developed separately for each matrix. We optimized three extraction parameters: (I) ratio of matrix to extracting solvents, (II) extraction temperature (on ice *vs.* room temperature), and (III) extract-transferred methods, *e.g.,* micropipette, microsyringe, and positive-displacement pipette. In summary, we found that extraction of approximately 60 mg of a fecal sample on ice with a mixture of water, acidified water (pH = 2), and diethyl ether (DE) at a ratio of 8:3:10 was the most effective and practical condition (Fig. 1C). We found that two cycles of extraction (1000 µL DE each) were sufficient to recover the target SCFAs. Despite following a previously published procedure [25] that recommended extracting the mixture for at least 15 min, we found that manually homogenizing feces before extraction using centrifugation produced comparable recoveries. We also found that dehydration of the sample extracts with Na_2_SO_4_ was necessary to prolong the GC-TOFMS lifetime, and hence we included this step even though it increased the sample-preparation time and had no effect on the method’s recoveries. The highest recoveries (Table S2) were achieved by employing a positive-displacement pipette in conjunction with the other optimal conditions (Fig. 1D), owing to the DE's low boiling point (34 °C). While the recoveries achieved with a microsyringe were similar to those obtained with a displacement pipette, its lower precision rendered it unsuitable for high-throughput analysis.

A procedure for extracting plasma SCFAs was fine-tuned by optimizing the number of extractions, acidity, and volume of the extraction solvent (Figs. 1C, 1D, and Table S2). We refined the extraction procedure at low temperature conditions. Each step was carried out on ice or under controlled temperature conditions to avoid the dissipation of SCFAs. The plasma samples underwent two rounds of extraction using plasma, DE and acidified water at a ratio of 10:20:1, as shown in Fig. 1D. This approach proved to be the most effective, resulting in good recoveries of the IS (Table S2).

For quantitative analysis, we found significant matrix effects (Table 1) on the fecal and plasma SCFA quantifications; therefore, the matrix-matched calibration method [32] was used to obtain an improved recovery. Finally, we evaluated the method’s recovery through a spike-in study using SCFA reference standards in both matrices. Overall, the average recoveries of the 13 SCFAs ranged from 73% ± 5% to 88% ± 2% (N = 3) in fecal matrices and from 93% ± 19% to 134% ± 18% (N = 3) in the plasma matrices (Table 1). The limit of detection (LOD) and limit of quantification (LOQ) ranged from 5 to 50 ng/mL and 14 to 152 ng/mL, respectively, for fecal samples, and from 0.007 to 5.14 µM and 0.024 to 17.13 µM for plasma samples (Table 1). The reproducibility was assessed by spiking a standard SCFA mixture at 20 µg in feces (60 mg) and 0.5 µg in plasma (100 µL). The percent relative standard deviation (%RSD) of peak areas ranged from 0.0% to 6.0% for intraday precision and from 2% to 17% for interday precision. (Table 1). To process one sample, the method takes approximately 10 min for sample preparation and 13.3 min for the GC-TOFMS analysis, suitable for high-throughput research and large-cohort studies.

### 3.2 Cohort characteristics and dietary record

Table 2 provides demographic characteristics of the two cohorts of healthy individuals. Combined, the two cohorts consisted of 59 males (37.6%) and 98 females (62.4%), with an average age of 23 years, ranging from 18 to 59 years. The majority of the participants (60.5%) had a normal body mass index (18.5–22.9 kg/m^2^), 31.2% were overweight (≥23 kg/m^2^), and 8.3% were underweight (<18.5 kg/m^2^). Most of the participants (45.2%) had a moderate exercise habit (1–149 mins/week), 28.7% had a regular exercise habit (≥150 min/week), and 26.1% had a sedentary lifestyle. On average, 47.6%, 18.4%, and 33.5% of the caloric distributions were from carbohydrates, proteins, and fats, respectively. Average levels of blood glucose (83.0 ± 5.0 mg/dL), cholesterol (188.0 ± 24.3 mg/dL), and triglycerides (65.0 ± 22.5 mg/dL) fell within the normal ranges of 77–99 mg/dL, < 200 mg/dL, and < 150 mg/dL, respectively.

**Table 2:** Demographic and clinical data of the study population.

Characteristics	Cohort 2019 (N = 60)	Cohort 2022 (N = 97)	Cohort 2019 + 2022 (N = 157)
**Age**	22.5 (19−57)	24 (18−59)	23 (18−59)
**Gender**			
Male	26 (43.3%)	33 (34.0%)	59 (37.6%)
Female	34 (56.7%)	64 (66.0%)	98 (62.4%)
**BMI**			
Underweight (< 18.5)	6 (10.0%)	7 (7.2%)	13 (8.3%)
Healthy weight (18.5 - 22.9)	30 (50.0%)	65 (67.0%)	95 (60.5%)
Overweight (≥ 23)	24 (40.0%)	25 (25.8%)	49 (31.2%)
**Physical activity**			
No exercise	18 (30.0%)	27 (27.8%)	45 (28.7%)
1-149 min/week	26 (43.3%)	45 (46.4%)	71 (45.2%)
≥ 150 min/week	16 (26.7%)	25 (25.8%)	41 (26.1%)
**Dietary Analysis**			
%Caloric distribution from Carbohydrate	50.1 ± 5.9	45.7 ± 6.7	47.6 ± 6.7
% Caloric distribution from Protein	17.7 ± 3.4	18.9 ± 3.5	18.4 ± 3.5
% Caloric distribution from Fat	31.5 ± 4.1	33.9 ± 4.8	33.5 ± 4.6
**Blood Chemistry Biomarker**			
Fasting blood glucose (mg/dL)	82.0 ± 4.9	84.0 ± 4.9	83.0 ± 5.0
Cholesterol (mg/dL)	193.0 ± 23.4	181.5 ± 26.3	188.0 ± 24.3
Triglyceride (mg/dL)	64.0 ± 23.7	68.0 ± 21.9	65.5 ± 22.5
Creatinine (mg/dL)	-	0.74 ± 0.12	-
Thai eGFR Equation (mL/min/1.73 m^*2^)	-	116.0 ± 15.0	-
eGFR MDRD (Caucasian; mL/min/1.73 m^*2^)	-	99.7 ± 11.1	-
eGFR CKD-EPI (mL/min/1.73 m^*2^)	-	114.8 ± 9.9	-
SGPT (U/L)	-	13.0 ± 4.0	-

BMI, body mass index; CKD-EPI, Chronic Kidney Disease Epidemiology Collaboration; eGFR, estimated glomerular filtration rate; MDRD, Modification of Diet in Renal Disease; SGPT, serum glutamic-pyruvic transaminase

The summary of daily nutrient intake is provided in Table S5 (data are presented as median ± median absolute deviation [MAD]). Overall, amounts of dietary fiber, crude fiber, animal protein, fat, vitamin B1, and iron showed no significant differences (*p* > 0.05) between females and males. On the other hand, daily energy, carbohydrate, protein, and vegetable protein intakes were significantly higher (*p* < 0.05) in males compared to females. Vitamin A (p-value < 0.005), B3 (p-value < 0.005), B2 (p-value < 0.0001), and calcium (p-value < 0.0001) were significantly higher in males, whereas for Vitamin C (p-value <0.05), females had higher levels than males.

Nonetheless, all the participants consumed lower carbohydrate, higher protein, moderate fat, and lower crude and dietary fiber than the Thai dietary reference intake values [36].

### 3.3 Concentrations of fecal and plasma SCFAs

**Fig. 2:**
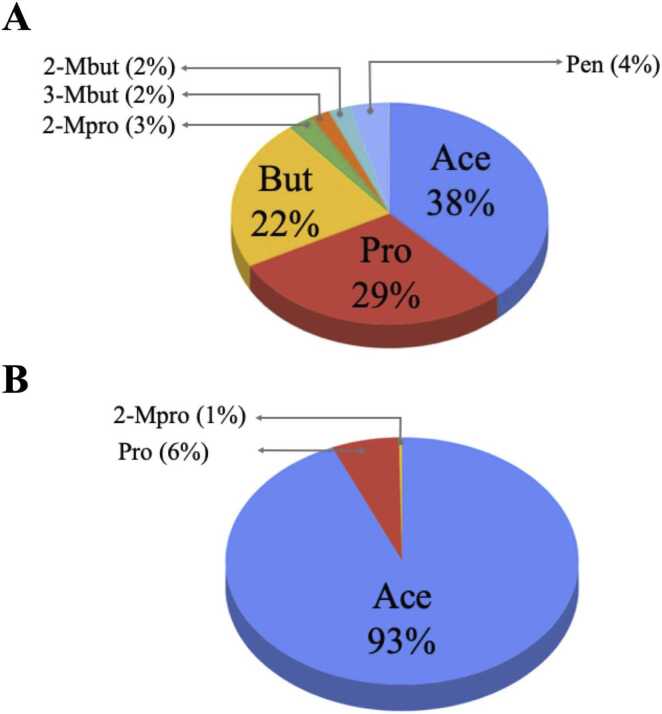
SCFA composition in fecal (A) and plasma (B) of healthy individuals (cohort 2019 + cohort 2022; N = 157) *Full detail is given in Table S6 (for fecal SCFAs) and Table S7 (for plasma SCFAs).

**Fig. 3:**
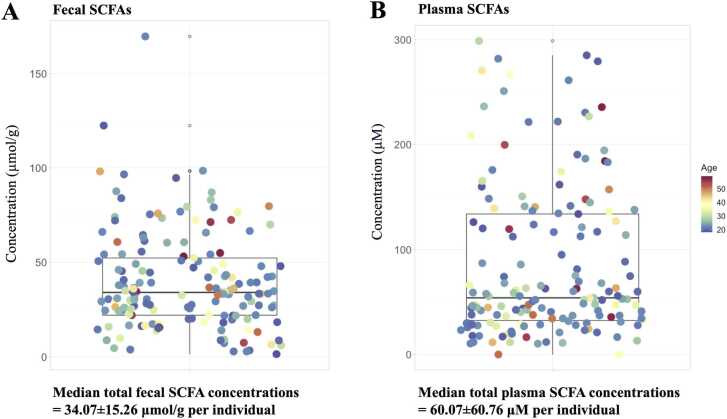
Total SCFA content of healthy individuals (cohort 2019 + cohort 2022; N = 157) in fecal samples (A) and plasma samples (B) labelled by age on a continuous scale. The boxplots represent the total SCFA concentration in median ± median absolute deviation (MAD). Full detail can be seen in Table S6 (for fecal SCFAs) and Table S7 (for plasma SCFAs).

**Fig. 4:**
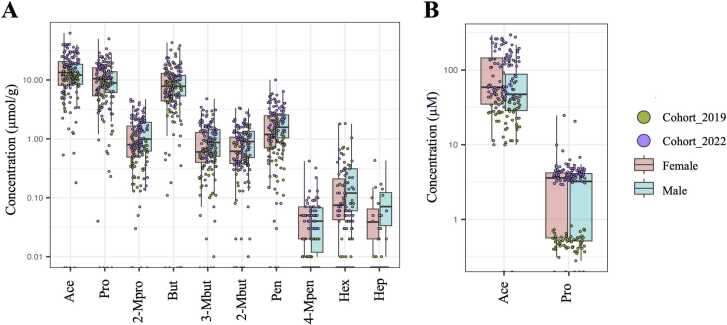
The individual SCFAs of healthy individuals (cohort 2019 + cohort 2022; N = 157) in fecal (A) samples and (B) plasma samples labeled by cohort. Full detail can be seen in Table S6 (for fecal SCFAs) and Table S7 (for plasma SCFAs). Separated SCFAs composition for cohort 2019 and cohort 2022 can be seen in Fig. S1.

The fecal and plasma SCFA concentrations are reported as median ± MAD, unless otherwise stated. Overall, 89% of total fecal SCFAs were the major SCFAs, consisting of Ace (12.61 ± 6.45 µmol/g), Pro (9.61 ± 5.09 µmol/g), and But (7.27 ± 4.42 µmol/g) (Fig. 2A). The minor fecal SCFAs were 2-Mpro (0.86 ± 0.66 µmol/g), 3-Mbut (0.64 ± 0.48 µmol/g), 2-Mbut (0.60 ± 0.44 µmol/g), and Pen (1.32 ± 0.94 µmol/g) (Fig. 2A). Only Ace (51.08 ± 49.46 µM) was a predominant SCFA in plasma samples, accounting for 93%, whereas Pro (3.41 ± 2.07 µM) and 2-Mpro (0.21 ± 0.66 µM) were minor SCFAs (Fig. 2B). The median total SCFA concentrations in feces and plasma of healthy individuals were 34.07 ± 15.26 µmol/g (Fig. 3A) and 60.01 ± 45.87 µM (Fig. 3B), respectively. Next, we investigated whether the concentration of total SCFAs was related to age and gender. We found no clear relationship between SCFA content and age in either fecal or plasma samples (Figs. 3A and 3B). Relationships between gender and SCFA concentrations showed similar patterns, with no significant differences seen in the individual fecal SCFA concentrations between males and females (Figs. 4A and 4B). The levels of total plasma SCFAs, Ace and Pro, concentrations collected in 2022, however, were significantly higher than those collected in 2019 (Fig. S2B).

### 3.4 Correlation analysis of SCFAs with other factors

#### 3.4.1 The correlation among fecal SCFAs

As seen in Fig. S3, most fecal SCFAs were significantly and positively correlated with each other. Eight SCFA pairs showed strong correlation coefficients ranging from 70% to 97%: Ace and Pro (85%), Ace and But (78%), Pro and But (70%), 2-Mpro and 3-Mbut (97%), 2-Mpro and 2-Mbut (84%), 2-Mpro and Pen (75%), 3-Mbut and 2-Mbut (89%), and 3-Mbut and Pen (71%). In contrast, the correlations between Hex and Ace, Hex and Pro, and Hex and But were slightly negative.

#### 3.4.2 The correlation between SCFAs and nutrients

**Fig. 5:**
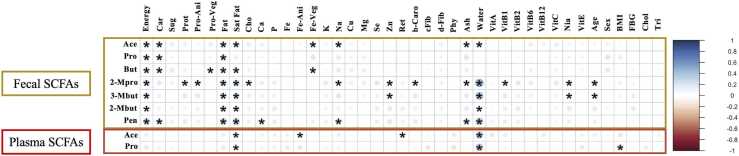
Spearman correlation coefficients between SCFAs and recorded dietary nutrients. Darker color and larger size of the dots represent higher correlation coefficient values. Red color indicates positive correlation, while blue color indicates negative correlation, and the absence of color indicates no correlation. Significant correlations are indicated with an asterisk (*). Abbreviations: “Ener” – Energy, “Car” – Carbohydrate, “Sug” – Sugar, “Prot” – Protein, “Pro-Ani” – Protein from animal, “Pro-Veg” – Protein from vegetable, “Fat” – Fat, “Sat Fat” – Saturated fat, “Cho” – Cholesterol, “Ca” – Calcium, “P” – Phosphorus, “Fe” – Iron, “Fe-Ani” – Iron from animal, “Fe-Veg” – Iron from vegetable, “K” – Potassium, “Na” – Sodium, “Cu” – Copper, “Mg” – Magnesium, “Se” – Selenium, “Zn” – Zinc, “Ret” – Retinol, “b-Caro” – beta-carotene, “cFib” – Crude fiber, “dFib” – Dietary fiber, “Phy” – Phytate, “Ash” – Ash, “Water” – water, “Vit” – Vitamin, “Nia” – Niacin, “FBG” – Fasting blood glucose, “Chol” – Blood cholesterol level, “Tri” – Blood triglyceride level.

The correlations between nutrients and SCFAs were determined (Fig. 5). Fecal Ace concentrations exhibited eight significantly positive correlations (*p* < 0.05) with energy, carbohydrate, fat, saturated fat, iron from vegetable, sodium, ash, and water. Fecal Pro concentrations were positively and significantly correlated with energy, carbohydrate, and fat. Fecal But concentrations were positively and significantly correlated with energy, carbohydrate, protein from vegetable, fat, saturated fat, and iron from vegetable. Fecal 2-Mpro, 3-Mbut, 2-Mbut, and Pen showed thirteen, six, four, and eight positive significant correlations, respectively. Mineral and vitamin, calcium, iron from vegetable, sodium, zinc, b-carotene, vitamin B1, and niacin exhibited significant positive correlations with fecal SCFAs. On the other hand, plasma Ace levels were positively and significantly correlated with saturated fat and water but were negatively correlated with iron from animal and vitamin A (retinol) (Fig. 5). Plasma Pro exhibited two positively significant correlations with saturated fat and water.

#### 3.4.3 The correlation between SCFAs and other determinants

Fig. 3 shows no differences in total fecal SCFAs and plasma SCFAs among ages. Although, we observed significant difference in fecal 2-Mpro and 3-Mbut concentrations among ages (Fig. 5), the sum of 2-Mpro and 3-Mbut accounted for only 5% of total fecal SCFAs (Fig. 2A). Thus, we concluded that SCFAs are independent of age. Other determinants, such as sex, BMI, fasting blood glucose, cholesterol, and triglyceride levels, do not show any significant correlations with fecal and plasma SCFAs, except for plasma Pro that was significantly negative correlated with BMI.

## 4 Discussion

In this study, we developed a derivatization-free GC-TOFMS method for quantifying fecal and plasma SFCAs and applied them to a cohort study. The DB-FFAP column, which is an acid-modified WAX, was shown to be suitable for the GC separation of non-derivatized SCFAs, especially in samples that contain acidic impurities like fecal samples. The sensitivity of our methods based on LOD values was approximately 5 times (for fecal matrix) and 26 times (for plasma matrix) better than those reported previously [25]. The high sensitivity of the methods offers a great opportunity to quantify trace levels of SCFAs, particularly in plasma samples. Typically, a method precision of less than 15% RSD from both intraday and interday tests should be achieved for the validation [37], [38]. However, intraday and interday variations found in plasma Hep were greater than 15% RSD (Table S4), probably owing to the target SCFA being present at trace levels only. Because the SCFAs have distinct chemical structures, we examined method efficiency by spiking tests with all authentic SCFA standards. Percent method recoveries for most SCFAs fell within the acceptable range of 80% − 120%, except for Ace in fecal matrix which had a recovery of 73%. The low recovery of Ace was possibly due to loss during evaporation or from partial partitioning into the aqueous layer. Compared to other SCFAs, Ace is more polar and soluble in water due to its relatively small molecular size. This property poses a challenge for recovering Ace from a fecal matrix in this and previous studies [39], [40], [41]. In summary, our method is rapid, sensitive, and precise. The overall protocols for feces and plasma analysis need only 10 and 13.3 min for sample preparation and GC-TOFMS analysis time, which is a marked improvement on methods used in previous studies [42], [43], [44], [45].

The quantification of fecal and plasma SCFAs of healthy individuals from the cohorts 2019 and 2022 (N = 157) revealed that the numbers of detected SCFAs from the two cohorts were similar but total concentrations were different. The comparable numbers of SCFAs detected from the two cohorts are likely due to methodological detection and biological similarity *e.g.*, the similar ethnic backgrounds, lifestyles, food cultures, and health conditions. Compared to the 2019 cohort, the 2022 cohort had higher levels of total SCFA in both feces and plasma (Fig. S2), a difference most likely resulting from the 2022 cohort's higher energy and dietary fiber intake (Fig. S4). Diet influences the composition of the gut microbiota which in turn impacts on SCFAs; for example, higher amounts of dietary fiber can increase the abundance of bacteria that produce SCFAs [46], [47]. In addition, significant individual differences in SCFA concentrations could be due to biological differences influenced by myriad factors such as dynamics of the human microbiome and dietary consumption [12], [48].

In fecal samples, the majority of SCFAs (89%) were Ace, Pro, and But, which is consistent with previous studies from various nations [18], [19], [20]. However, the Thai population (N = 157) had much lower fecal concentrations of the three major SCFAs than the British population (N = 3), which in turn had significantly lower fecal concentrations than the Malaysian population (N = 50) (Fig. S5). On the other hand, Ace was identified as the most prevalent SCFA in plasma, accounting for 93% of the total SCFAs present. This showed that plasma SCFAs levels in this study were similar to the study of UK individuals (N = 3). Colonocytes utilize specific segments of SCFAs for energy, transporting the remaining portions to the circulation and liver tissue [49]. Although hepatocytes generally use SCFAs as their primary source of energy, they do not metabolize Ace, and as a result, Ace remains in the systemic circulation [50], [51]. The average plasma Ace concentration (84 µM) from our study was comparable to that reported in a study of healthy individuals in the UK (67 µM, N = 3, Fig. S5).

Previous studies have focused on the three main fecal SCFAs, namely Ace, Pro, and But, while the branched SCFAs have been less explored. The concentration of these branched SCFAs could have an impact on both microbe and host metabolisms. For example, increased levels of branched SCFAs (a subset of branched-chain fatty acids, BCFAs[52]), may indicate a shift from carbohydrate to protein fermentation [53], which occurs when carbohydrate fermentation is limited [54]. Additionally, increased protein consumption was positively associated with the production of BCFAs, including isobutyrate and isovalerate in people with a body mass index of 40 or above [53]. Thus, to obtain information in this under-studied area to help broaden research in human health and disease, we quantified fecal branched SCFAs, including 2-Mpro, 3-Mbut, 2-Mbut, and 4-Mpen (Fig. 4).

To investigate the association of SCFAs with age and sex, we combined data from both cohorts for the analysis to encompass a wider population. Our analysis revealed no significant age- and sex-related differences in fecal and plasma SCFA concentrations (Figs. 3 and 4). Several factors may explain the lack of the associations of SCFA concentrations and age or sex. In this context, we observed insignificant differences in food consumption and nutrient intakes between males and females (Table S5), particularly fiber and crude fiber, which are known to affect SCFA production [55]. All the participants were in healthy condition, and not exposed to factors that could significantly alter the gut environment, such as prolonged antibiotic use, alcohol consumption, or significant dietary changes. In addition, most of the participants in this study were university students residing in Bangkok, and consequently they tended to have similar lifestyles and were exposed to the same environment. These factors may have contributed to the insignificant correlation of SCFAs concentrations with sex or age. Thus, it might not be necessary to design age- and sex-matching-based studies of SCFAs in clinical research, which can be difficult. However, more quantitative data on these metabolites from different locations and populations with reliable and well-defined methods is needed. Although this could be very challenging, levels of SCFAs in healthy people from free-living or well-controlled human studies are regarded as a rich resource for future physiology and biomarker research, and more research is needed in this area.

Numerous studies in humans have found that fiber-rich or prebiotic-fiber-supplemented diets promote healthy gut microbiota, diversity, bacterial growth, and SCFA production [46]. However, it's important to note that this study did not specifically design many critical parameters to address this question, nor was it the primary objective. The results we discuss here are just observational and correlative results, which may be useful in guiding future studies. We examined the correlations of SCFAs and diets. We found that 17 nutrients were significantly correlated with fecal SCFAs and 4 nutrients significantly correlated with plasma SCFAs. These nutrients, such as Car, Prot, and Fat, are often the main sources of body energy and microbial energy metabolism [56], [57]. Other nutrients, such as Fe, Na, Zn, b-Caro, Ash, Vit B1, and Nia, were not widely explored in terms of possible correlations with SCFA levels. In addition to those nutrients, earlier research has shown that water sources and levels of water consumption affect the gut microbial community. Vanhaecke and co-workers reported higher levels of Campylobacter and lower levels of Bacteroides, Odoribacter, and Streptococcus in well-water drinkers compared to low-water drinkers [58]. Water is necessary to preserve the structure and functionality of microbial cells. It also helps to dilute intracellular toxic substances and protects them from oxidative and electrostatic field damage[59]. As shown in Fig. 5, levels of water consumption showed positively significant correlations with fecal Ace, 2-Mpro, 3-Mbut, 2-Mbut, and Pen; and plasma Ace and Pro. This suggests that water consumption could have an impact on shaping gut microbiota and SCFA production.

## 5 Conclusion

We developed and validated a simple, fast, and accurate derivatization-free GC-TOFMS method for quantifying six linear- and seven branched-SCFAs in feces and plasma samples. We quantified the total SCFAs in feces and plasma of healthy individuals (N = 157) and reported the absolute concentrations. Our findings show that the concentration of SCFAs in these 157 healthy individuals are age- and sex-independent. The results here can be used as a resource for future research on gut microbiome, metabolomics, and biomarker discovery.

## 6 Limitations

One limitation of this study was that the dietary records may be biased because they are obtained from individual’s estimate of personal consumption rather than on experimental measurement. In a future study, a controlled diet study should be conducted to account for confounding factors caused by diets. Another limitation was that our subjects were mainly university students aged between 18–22 years old (28% of the whole population, while the remaining subjects were aged from 23–60 years. Ideally, studies such as this should recruit individuals with ages more equally distributed among 18–60 years, where the 18–22 years group should consist of approximately 12% of all subjects. However, it is also challenging to recruit healthy subjects covering all age groups as elderly subjects are more prone to have medical problems.

## Ethics approval and consent to participate

All the experiment and sample collection protocols were approved by Institutional Review Board of the Faculty of Medicine, Chulalongkorn University, Bangkok, Thailand (IRB No. 057/62 for cohort 2019 and IRB No. 372/64 for cohort 2022) and conducted in compliance with the Helsinki Declaration. Fecal and blood plasma samples were collected from healthy individuals with informed consent from all subjects.

## Funding

This research project is supported by Mahidol University (to SK), Bangkok, Thailand. This research has received funding support from the NSRF *via* the Program Management Unit for Human Resources & Institutional Development, Research and Innovation [grant number B05F650015] (to SK), the National Research Council of Thailand (10.13039/100012153NRCT) and Mahidol University (N42A650370, to NJ), and Charoen Pokphand Foods, Bangkok, Thailand (to SK). This research was supported by a Siriraj Graduate Scholarship of the Faculty of Medicine Siriraj Hospital, Mahidol University awarded to WM. This project was partially supported by the Research Excellence Development (10.13039/100011115RED) Program of the Faculty of Medicine, Siriraj Hospital, 10.13039/501100004156Mahidol University.

## CRediT authorship contribution statement

**Weerawan Manokasemsan:** Conceptualization, Formal analysis, Investigation, Methodology, Project administration, Data curation, Visualization, Writing – original draft, Writing – review & editing. **Narumol Jariyasopit:** Conceptualization, Methodology, Formal analysis, Visualization, Writing - original draft, Writing – review & editing. **Patcha Poungsombat:** Methodology, Formal analysis, Visualization. **Khwanta Kaewnarin:** Methodology, Data curation, Project administration, Formal analysis, Writing – review & editing. **Kwanjeera Wanichthanarak:** Formal analysis, Visualization, Writing – review & editing. **Alongkorn Kurilung:** Methodology, Data curation, Project administration. **Kassaporn Duangkumpha:** Methodology, Writing – review & editing. **Suphitcha Limjiasahapong:** Methodology. **Yotsawat Pomyen:** Data curation, Writing – review & editing. **Roongruedee Chaiteerakij:** Methodology, Investigation, Data curation. **Rossarin Tansawat:** Methodology, Investigation, Data curation, Writing – review & editing. **Chatchawan Srisawat:** Funding acquisition, Writing – review & editing. **Yongyut Sirivatanauksorn:** Conceptualization, Funding acquisition, Resources. **Vorapan Sirivatanauksorn:** Conceptualization, Funding acquisition, Resources. **Sakda Khoomrung:** Conceptualization, Investigation, Methodology, Funding acquisition, Resources, Data curation, Formal analysis, Supervision, Writing – original draft, Writing – review & editing.

## Declaration of Competing Interest

The authors declare that they have no competing interest with the contents of this article.

## Data Availability

This article contains supporting information in supplementary table and figures. The data will be made available on request.

## References

[1] Group N.H.W., Peterson J., Garges S., Giovanni M., McInnes P., Wang L. (2009). The NIH human microbiome project. Genome Res.

[2] Qin J., Li R., Raes J., Arumugam M., Burgdorf K.S., Manichanh C. (2010). A human gut microbial gene catalogue established by metagenomic sequencing. Nature.

[3] Weersma R.K., Zhernakova A., Fu J. (2020). Interaction between drugs and the gut microbiome. Gut.

[4] Singh R.K., Chang H.W., Yan D., Lee K.M., Ucmak D., Wong K. (2017). Influence of diet on the gut microbiome and implications for human health. J Transl Med.

[5] Zhang S., Chen D.C. (2019). Facing a new challenge: the adverse effects of antibiotics on gut microbiota and host immunity. Chin Med J (Engl).

[6] Lewis G., Wang B., Shafiei Jahani P., Hurrell B.P., Banie H., Aleman Muench G.R. (2019). Dietary fiber-induced microbial short chain fatty acids suppress ILC2-dependent airway inflammation. Front Immunol.

[7] Corrêa-Oliveira R., Fachi J.L., Vieira A., Sato F.T., Vinolo M.A.R. (2016). Regulation of immune cell function by short-chain fatty acids. Clin Transl Immunol.

[8] Parada Venegas D., De la Fuente M.K., Landskron G., Gonzalez M.J., Quera R., Dijkstra G. (2019). Short chain fatty acids (SCFAs)-mediated gut epithelial and immune regulation and its relevance for inflammatory bowel diseases. Front Immunol.

[9] van der Hee B., Wells J.M. (2021). Microbial regulation of host physiology by short-chain fatty acids. Trends Microbiol.

[10] Correa-Oliveira R., Fachi J.L., Vieira A., Sato F.T., Vinolo M.A. (2016). Regulation of immune cell function by short-chain fatty acids. Clin Transl Immunol.

[11] Yao Y., Cai X., Fei W., Ye Y., Zhao M., Zheng C. (2022). The role of short-chain fatty acids in immunity, inflammation and metabolism. Crit Rev Food Sci Nutr.

[12] Koh A., De Vadder F., Kovatcheva-Datchary P., Bäckhed F. (2016). From dietary fiber to host physiology: short-chain fatty acids as key bacterial metabolites. Cell.

[13] Dalile B., Van Oudenhove L., Vervliet B., Verbeke K. (2019). The role of short-chain fatty acids in microbiota-gut-brain communication. Nat Rev Gastroenterol Hepatol.

[14] Aho V.T.E., Houser M.C., Pereira P.A.B., Chang J., Rudi K., Paulin L. (2021). Relationships of gut microbiota, short-chain fatty acids, inflammation, and the gut barrier in Parkinson’s disease. Mol Neurodegener.

[15] Pozuelo M., Panda S., Santiago A., Mendez S., Accarino A., Santos J. (2015). Reduction of butyrate- and methane-producing microorganisms in patients with Irritable Bowel Syndrome. Sci Rep-Uk.

[16] Vatanen T., Franzosa E.A., Schwager R., Tripathi S., Arthur T.D., Vehik K. (2018). The human gut microbiome in early-onset type 1 diabetes from the TEDDY study. Nature.

[17] Chen H., Meng L., Shen L. (2022). Multiple roles of short-chain fatty acids in Alzheimer disease. Nutrition.

[18] Boets E., Gomand S.V., Deroover L., Preston T., Vermeulen K., De Preter V. (2017). Systemic availability and metabolism of colonic-derived short-chain fatty acids in healthy subjects: a stable isotope study. J Physiol.

[19] Huda-Faujan N., Abdulamir A.S., Fatimah A.B., Anas O.M., Shuhaimi M., Yazid A.M. (2010). The impact of the level of the intestinal short chain Fatty acids in inflammatory bowel disease patients versus healthy subjects. Open Biochem J.

[20] Lotti C., Rubert J., Fava F., Tuohy K., Mattivi F., Vrhovsek U. (2017). Development of a fast and cost-effective gas chromatography-mass spectrometry method for the quantification of short-chain and medium-chain fatty acids in human biofluids. Anal Bioanal Chem.

[21] Blaak E.E., Canfora E.E., Theis S., Frost G., Groen A.K., Mithieux G. (2020). Short chain fatty acids in human gut and metabolic health. Benef Microbes.

[22] Zhang S., Wang H., Zhu M.J. (2019). A sensitive GC/MS detection method for analyzing microbial metabolites short chain fatty acids in fecal and serum samples. Talanta.

[23] Gao X., Pujos-Guillot E., Martin J.F., Galan P., Juste C., Jia W. (2009). Metabolite analysis of human fecal water by gas chromatography/mass spectrometry with ethyl chloroformate derivatization. Anal Biochem.

[24] Zheng X., Qiu Y., Zhong W., Baxter S., Su M., Li Q. (2013). A targeted metabolomic protocol for short-chain fatty acids and branched-chain amino acids. Metabolomics.

[25] Han X., Guo J., You Y., Yin M., Ren C., Zhan J. (2018). A fast and accurate way to determine short chain fatty acids in mouse feces based on GC-MS. J Chromatogr B Anal Technol Biomed Life Sci.

[26] Zhao G., Nyman M., Jonsson J.A. (2006). Rapid determination of short-chain fatty acids in colonic contents and faeces of humans and rats by acidified water-extraction and direct-injection gas chromatography. Biomed Chromatogr.

[27] Garcia-Villalba R., Gimenez-Bastida J.A., Garcia-Conesa M.T., Tomas-Barberan F.A., Carlos Espin J., Larrosa M. (2012). Alternative method for gas chromatography-mass spectrometry analysis of short-chain fatty acids in faecal samples. J Sep Sci.

[28] Dave M., Higgins P.D., Middha S., Rioux K.P. (2012). The human gut microbiome: current knowledge, challenges, and future directions. Transl Res.

[29] Todd, J. C., 1874–1928, Sanford, A. H., 1882–1959, Davidsohn, I., 1895–1979, Henry, J. B., 1928–2009, and Todd, J. C., 1874–1928. (1979) *Clinical diagnosis and management by laboratory methods / Todd - Sanford - Davidsohn.*, Philadelphia: Saunders, United States.

[30] Khoomrung S., Chumnanpuen P., Jansa-Ard S., Ståhlman M., Nookaew I., Borén J. (2013). Rapid quantification of yeast lipid using microwave-assisted total lipid extraction and HPLC-CAD. Anal Chem.

[31] Dunn W.B., Erban A., Weber R.J.M., Creek D.J., Brown M., Breitling R. (2013). Mass appeal: metabolite identification in mass spectrometry-focused untargeted metabolomics. Metabolomics.

[32] Limjiasahapong S., Kaewnarin K., Jariyasopit N., Hongthong S., Nuntasaen N., Robinson J.L. (2020). UPLC-ESI-MRM/MS for Absolute Quantification and MS/MS Structural Elucidation of Six Specialized Pyranonaphthoquinone Metabolites From Ventilago harmandiana. Front Plant Sci.

[33] Wanichthanarak K., In-On A., Fan S., Fiehn O., Wangwiwatsin A., Khoomrung S. (2024). Data processing solutions to render metabolomics more quantitative: case studies in food and clinical metabolomics using Metabox 2.0. Gigascience.

[34] (2019) INMUCAL. Institute of Nutrition, Mahidol University.

[35] Kaewnarin K., Limjiasahapong S., Jariyasopit N., Anekthanakul K., Kurilung A., Wong S.C.C. (2021). High-resolution QTOF-MRM for highly accurate identification and quantification of trace levels of triterpenoids in ganoderma lucidum mycelium. J Am Soc Mass Spectr.

[36] Ivanovitch K., Klaewkla J., Chongsuwat R., Viwatwongkasem C., Kitvorapat W. (2014). The intake of energy and selected nutrients by thai urban sedentary workers: an evaluation of adherence to dietary recommendations. J Nutr Metab.

[37] Mishra P., Albiol-Chiva J., Bose D., Durgbanshi A., Peris-Vicente J., Carda-Broch S. (2018). Optimization and validation of a chromatographic method for the quantification of isoniazid in urine of tuberculosis patients according to the european medicines agency guideline. Antibiot (Basel).

[38] Tiwari G., Tiwari R. (2010). Bioanalytical method validation: an updated review. Pharm Methods.

[39] Rohde J.K., Fuh M.M., Evangelakos I., Pauly M.J., Schaltenberg N., Siracusa F. (2022). A gas chromatography mass spectrometry-based method for the quantification of short chain fatty acids. Metabolites.

[40] Kim H., Kwon J., Choi S.Y., Ahn Y.G. (2019). Method development for the quantitative determination of short chain fatty acids in microbial samples by solid phase extraction and gas chromatography with flame ionization detection. J Anal Sci Technol.

[41] García-Villalba R., Giménez-Bastida J.A., García-Conesa M.T., Tomás-Barberán F.A., Carlos Espín J., Larrosa M. (2012). Alternative method for gas chromatography-mass spectrometry analysis of short-chain fatty acids in faecal samples. J Sep Sci.

[42] Zhang C., Fan L., Zhao H. (2020). Rapid detection of short-chain fatty acids in biological samples. Chromatographia.

[43] Kim K.S., Lee Y., Chae W., Cho J.Y. (2022). An improved method to quantify short-chain fatty acids in biological samples using gas chromatography-mass spectrometry. Metabolites.

[44] Wang H., Liu Y., Shao J., Luo Y., Cai W., Chen L. (2020). Rapid and accurate simultaneous determination of seven short-chain fatty acids in feces by gas chromatography – mass spectrometry (GC-MS): application in type 2 diabetic rats and drug therapy. Anal Lett.

[45] Wang R., Fan C., Fan X., Zhao Y., Wang Y., Li P. (2020). A fast and accurate way to determine short chain fatty acids in human serum by GC–MS and their distribution in children with digestive diseases. Chromatographia.

[46] Birkeland E., Gharagozlian S., Valeur J., Aas A.-M. (2023). Short-chain fatty acids as a link between diet and cardiometabolic risk: a narrative review. Lipids Health Dis.

[47] Fu Y., Moscoso D.I., Porter J., Krishnareddy S., Abrams J.A., Seres D. (2020). Relationship between dietary fiber intake and short-chain fatty acid-producing bacteria during critical illness: a prospective cohort study. JPEN J Parent Enter Nutr.

[48] McOrist A.L., Abell G.C.J., Cooke C., Nyland K. (2008). Bacterial population dynamics and faecal short-chain fatty acid (SCFA) concentrations in healthy humans. Br J Nutr.

[49] Wu Y., Xu H., Tu X., Gao Z. (2021). The role of short-chain fatty acids of gut microbiota origin in hypertension. Front Microbiol.

[50] Silva Y.P., Bernardi A., Frozza R.L. (2020). The role of short-chain fatty acids from gut microbiota in gut-brain communication. Front Endocrinol (Lausanne).

[51] Yamashita H., Kaneyuki T., Tagawa K. (2001). Production of acetate in the liver and its utilization in peripheral tissues. Biochim Biophys Acta.

[52] Lu H., Wang Z., Cao B., Cong F., Wang X., Wei W. (2024). Dietary sources of branched-chain fatty acids and their biosynthesis, distribution, and nutritional properties. Food Chem.

[53] Rios-Covian D., González S., Nogacka A.M., Arboleya S., Salazar N., Gueimonde M. (2020). An overview on fecal branched short-chain fatty acids along human life and as related with body mass index: associated dietary and anthropometric factors. Front Microbiol.

[54] Yao C.K., Muir J.G., Gibson P.R. (2016). Review article: insights into colonic protein fermentation, its modulation and potential health implications. Aliment Pharmacol Ther.

[55] Tan J.K., Macia L., Mackay C.R. (2023). Dietary fiber and SCFAs in the regulation of mucosal immunity. J Allergy Clin Immunol.

[56] Singh P., Kesharwani R.K., Keservani R.K., Bagchi D. (2017). Sustained Energy for Enhanced Human Functions and Activity.

[57] den Besten G., van Eunen K., Groen A.K., Venema K., Reijngoud D.J., Bakker B.M. (2013). The role of short-chain fatty acids in the interplay between diet, gut microbiota, and host energy metabolism. J Lipid Res.

[58] Vanhaecke T., Bretin O., Poirel M., Tap J. (2022). Drinking water source and intake are associated with distinct gut microbiota signatures in us and uk populations. J Nutr.

[59] Hallsworth J.E. (2022). Water is a preservative of microbes. Micro Biotechnol.

