# Supplementary material for: Quantifying fecal and plasma short-chain fatty acids in healthy Thai individuals
Source: Comput Struct Biotechnol J. 2024 May 8;23:2163–72. doi: 10.1016/j.csbj.2024.05.007 (PMC11141283; doi:10.1016/j.csbj.2024.05.007)
Supplement: Supplementary file 1 — Supplementary material [file mmc1.docx]

**Figure legends**

**Supplementary figure legends**

**Figure S1:** Comparison of the total fecal and plasma SCFA profiles in (A-B) cohort 2019 and (C-D) cohort 2022.

**Figure S2**: The total (A) fecal and (B) plasma SCFA concentrations compared between cohort 2019 and cohort 2022 (*p < 0.05; **p < 0.01; ***p<0.001; ****p < 0.0001).

**Figure S3:** The correlations among fecal SCFAs. The value of the correlation coefficient represents the extent to which the corresponding data points for two variables, one SCFA on the x-axis and another SCFA on the y-axis, aligned closely to the line of the best fit. A darker color and larger size of the dots represent higher correlation coefficient values. Red color indicates positive correlation and blue indicates negative correlation.

**Figure S4:** The comparison of (A) consumption of dietary fiber and (B) energy content that calculated from dietary record compared between cohort 2019 and cohort 2022 (*p < 0.05; **p < 0.01; ***p<0.001; ****p < 0.0001).

**Figure S5:** Comparisons of (A) fecal and (B) plasma SCFA concentrations among Thai, UK, and Malaysian populations (*p < 0.05; **p < 0.01; ***p<0.001; ****p < 0.0001). Data can be observed in Table S8.
