# Supplementary material for: Quantifying fecal and plasma short-chain fatty acids in healthy Thai individuals
Source: Comput Struct Biotechnol J. 2024 May 8;23:2163–72. doi: 10.1016/j.csbj.2024.05.007 (PMC11141283; doi:10.1016/j.csbj.2024.05.007)
Supplement: Supplementary file 2 — Supplementary material [file mmc2.docx]

| **Supplementary Table S1. the targeted quantitative masses and qualitative masses of each analyte** | | | | | |  |
| --- | --- | --- | --- | --- | --- | --- |
| **Order** | **Metabolites** | **Retention time (min)** | **Q1** | **Q2** | **Ion Ratio** | **Ion Ratio Tolerance (%)** |
| 1 | Ace | 6.39 | 45.00 | 60.02 | 1.47 | 30 |
| 2 | Pro-d_5_ | 7.04 | 79.06 | 45.00 | 1.36 | 30 |
| 3 | Pro | 7.1 | 74.03 | 45.00 | 1.09 | 30 |
| 4 | 2-Mpro | 7.33 | 43.06 | 73.02 | 3.55 | 50 |
| 5 | But | 7.82 | 60.02 | 73.03 | 3.47 | 30 |
| 6 | 3-Mbut | 8.13 | 60.02 | 87.05 | 4.23 | 30 |
| 7 | 2-Mbut | 8.14 | 74.03 | 41.05 | 0.75 | 50 |
| 8 | Pen | 8.66 | 60.02 | 73.03 | 2.93 | 30 |
| 9 | 3-Mpen | 9.06 | 60.02 | 87.05 | 9.69 | 30 |
| 10 | 4-Mpen | 9.14 | 57.07 | 74.04 | 1.72 | 30 |
| 11 | Hex | 9.44 | 60.02 | 73.03 | 2.34 | 30 |
| 12 | 2-Mhex | 9.6 | 74.03 | 87.05 | 3.11 | 30 |
| 13 | 4-Mhex | 10.01 | 71.09 | 55.06 | 1.01 | 30 |
| 14 | Hep | 10.18 | 60.02 | 73.03 | 2.11 | 30 |
| scan mode with m/z 30-400 Da | | |  |  |  |  |
| The tolerance was set to 0.1 Da | |  |  |  |  |  |
| Q1 – Quantifier ion |  |  |  |  |  |  |
| Q2 – Quanlifier ion |  |  |  |  |  |  |
| Ace : Acetic acid; Pro-d_5_: Propionic-d_5_ acid (as internal standard); Pro: Propionic acid; 2-Mpro: 2- | | | | | |  |
| Methylpropionic acid; But: Butanoic acid; 3-Mbut: 3-Methylbutanoic acid; 2-Mbut: 2-Methylbutanoic | | | | | | |
| acid; Pen: Pentanoic acid; 3-Mpen: 3-Methylpentanoic acid; 4-Mpen: 4-Methylpentanoic acid; Hex: | | | | | | |
| Hexanoic acid; 2-Mhex: 2-Methylhexanoic acid; 4-Mhex: 4-Methylhexanoic acid; Hep: Heptanoic acid | | | | | | |

| **Supplementary Table S2. Extraction adjustment** | | | | | | | | | | | | | | |
| --- | --- | --- | --- | --- | --- | --- | --- | --- | --- | --- | --- | --- | --- | --- |
| **Experi-ment** | **%Recovery (N=3)** | **Transferring** | **Matrix** | **Sample Mass/Volume** | **5M HCl (µL)** | **MilliQ Water (µL)** | **No. of extract-**  **ion** | **DE amount (µL)** | **Combined supernatant** | **Shaking** | **On ICE** | **Na2SO4 Dehydrat-ion** | **[IS]**  (ng/uL) | **Centrifuge Temp (°C)** |
| DE1 | 33.43 ± 3.18 | Micropipette | In DE | - | 300 | 800 | 2 | 1000 + 1000 | Yes | No | No | No | 20 | 4 |
| DE2 | 73.9 ±23.45 | Micropipette | In DE | - | 300 | 800 | 2 | 1000 + 1000 | No | No | Yes | Yes | 20 | 4 |
| DE3 | 92 ±26.01 | Micropipette | In DE | - | 300 | 800 | 2 | 1000 + 1000 | No | Yes | Yes | No | 20 | 4 |
| FC1 | 13.33 ± 2.93 | Micropipette | In Feces | 300 mg | 300 | 800 | 2 | 1000 + 1000 | No | Yes | No | No | 20 | 4 |
| FC2 | 36.35 ± 29.63 | Micropipette | In Feces | 300 mg | 300 | 800 | 2 | 1000 + 1000 | No | No | No | No | 20 | 4 |
| FC3 | 35.34 ± 25.5 | Micropipette | In Feces | 300 mg | 300 | 800 | 1 | 2000 | No | No | No | No | 10 | 4 |
| FC4 | 39.97 ± 11.91 | Micropipette | In Feces | 300 mg | 300 | 800 | 1 | 2000 | No | No | No | Yes | 10 | 4 |
| FC5 | 41.8 ± 8.58 | Micropipette | In Feces | 100 mg | 300 | 800 | 1 | 2000 | No | No | No | Yes | 20 | 4 |
| FC6 | 42.77 ± 5.24 | Micropipette | In Feces | 60 mg | 300 | 800 | 1 | 2000 | No | No | No | Yes | 20 | 4 |
| FC7 | 37.53 ± 1.8 | Micropipette | In Feces | 30 mg | 300 | 800 | 1 | 2000 | No | No | No | Yes | 20 | 4 |
| DE4 | 92.97 ± 3.84 | Microsyringe | In DE |  | 300 | 800 | 2 | 1000 + 1000 | No | No | No | Yes | 10 | 4 |
| DE5 | 104.83 ± 13.59 | Microsyringe | In DE |  | 300 | 800 | 2 | 1000 + 1000 | Yes | No | Yes | No | 10 | -10 |
| FC8 | 106.63 ± 17.61 | Microsyringe | In Feces | 60mg | 300 | 800 | 2 | 1000 + 1000 | Yes | No | Yes | No | 10 | -10 |
| DE6 | 106.37 ± 5.64 | Positive Displacement pipette | In DE |  | 300 | 800 | 2 | 1000 + 1000 | Yes | No | Yes | No | 10 | -10 |
| FC9 | 89.21 ± 11.61 | Positive Displacement pipette | In Feces | 60mg | 300 | 800 | 2 | 1000 + 1000 | Yes | No | Yes | No | 10 | -10 |
| FC9r | 90.05 ± 5.69 | Positive Displacement pipette | In Feces | 60mg | 300 | 800 | 2 | 1000 + 1000 | Yes | No | Yes | No | 10 | -10 |
| DE7 | 92.4 ± 3.72 | Positive Displacement pipette | In DE | 100µL Water | No | - | 2 | 100+  100 | Yes | No | Yes | Yes | 10 | -10 |
| DE8 | 109.2 ± 11.36 | Positive Displacement pipette | In DE | 100µL Water | 10uL | - | 2 | 100+  100 | Yes | No | Yes | Yes | 10 | -10 |
| PM1 | 102.6 ± 12.26 | Positive Displacement pipette | In plasma | 100µL Plasma | 10 uL | - | 2 | 100 + 100 | Yes | No | Yes | Yes | 10 | -10 |
| DE9 | 89.1 ± 1.2 | Positive Displacement pipette | In DE | 100µL Water | 10 uL | - | 2 | 100+  100 | Yes | No | Yes | Yes | 10 | -10 |
| DE10 | 84.83 ± 10.56 | Positive Displacement pipette | In DE | 100µL Water | 10 uL | - | 2 | 100+  100 | Yes | No | Yes | Yes | 5 | -10 |
| PM2 | 116.48 ± 15.34 | Positive Displacement pipette | In DE | 100µL Plasma | 10 uL | - | 2 | 100+  100 | Yes | No | Yes | Yes | 5 | -10 |
| DE11 | 89.27 ± 20.57 | Positive Displacement pipette | In DE | 100µL Plasma | 10 uL | - | 2 | 200+  200 | Yes | No | Yes | Yes | 5 | -10 |
| PM3 | 87.53 ± 6.4 | Positive Displacement pipette | In plasma | 100µL Plasma | 10 uL | - | 2 | 200+  200 | Yes | No | Yes | Yes | 5 | -10 |
| PM3r | 93.50 ± 1.87 | Positive Displacement pipette | In plasma | 100µL Plasma | 10 uL | - | 2 | 200+  200 | Yes | No | Yes | Yes | 5 | -10 |
| DE12 | 120.8 ± 26.1 | Positive Displacement pipette | In DE | 100µL Plasma | 10 uL | - | 2 | 300+  300 | Yes | No | Yes | Yes | 5 | -10 |
| PM4 | 85.33 ± 3.26 | Positive Displacement pipette | In plasma | 100µL Plasma | 10 uL | - | 2 | 300+  300 | Yes | No | Yes | Yes | 5 | -10 |
| For 'FC9r' and 'PM3r', these conditions are the repetition of FC9 and PM3, respectively | | | | | | |  |  |  |  |  |  |  |  |

| **Supplementary Table S3. SCFAs extraction result in fecal and plasma matrix** | | | | | | | | | |
| --- | --- | --- | --- | --- | --- | --- | --- | --- | --- |
| **Sample** |  | **Standards** | **%Recovery (N=3)** | **Matrix effect (%)** | **Matrix-matched calibration curves** | **R^2^** | | | **%Precision from spiked recovery** |
| **Fecal Sample** | 1 | Ace | 73.36 ± 1.69 | 485 | y = 1.71x 10^7^ x – 3.36 x 10^7^ | 0.993 | | | 2.3 |
|  | 2 | *Pro-d5 | 85.36 ± 8.78 | - | y = 6.66x10^6^ x – 1.18x10^7^ | 0.99 | | | 0.3 |
|  | 3 | Pro | 83.34 ± 1.89 | 443 | y = 8.4x10^6^ x – 1.43x10^7^ | 0.991 | | | 2.3 |
|  | 4 | 2-Mpro | 83.21 ± 0.96 | 540 | y = 2.07x10^7^ x – 3.41x10^7^ | 0.992 | | | 1.2 |
|  | 5 | But | 88.13 ± 2.44 | 429 | y = 2.79x10^7^ x – 3.79x10^7^ | 0.994 | | | 2.8 |
|  | 6 | 3-Mbut | 85.55 ± 0.93 | 524 | y = 2.02x10^7^ x – 3.13x10^7^ | 0.991 | | | 1.1 |
|  | 7 | 2-Mbut | 80.06 ± 1.70 | 572 | y = 1.63x10^7^ x – 1.18x10^7^ | 0.992 | | | 2.1 |
|  | 8 | Pen | 75.98 ± 1.34 | 600 | y = 2.84x10^7^ x – 5.07x10^7^ | 0.991 | | | 1.8 |
|  | 9 | 3-Mpen | 77.49 ± 1.17 | 571 | y = 1.75x10^7^ x – 2.28x10^7^ | 0.992 | | | 1.5 |
|  | 10 | 4-Mpen | 75.79 ± 1.55 | 572 | y = 1.66x10^7^ x – 1.58x10^7^ | 0.99 | | | 2.0 |
|  | 11 | Hex | 81.01 ± 1.63 | 533 | y = 1.79x10^7^ x – 2.8x10^7^ | 0.991 | | | 2.0 |
|  | 12 | 2-Mhex | 72.79 ±1.17 | 583 | y = 2.12x10^7^ x – 2.2x10^7^ | 0.993 | | | 1.6 |
|  | 13 | 4-Mhex | 80.02 ± 1.78 | 572 | y = 2.13x10^7^ x – 1.15x10^7^ | 0.995 | | | 2.2 |
|  | 14 | Hep | 75.38 ± 0.87 | 564 | y = 1.63x10^7^ x – 2.25x10^7^ | 0.992 | | | 1.2 |
| **Plasma Sample** | 1 | Ace | 92.85 ± 18.64 | 135 | y = 2.21x10^8^ x + 2.96x10^7^ | 0.998 | | |  |
|  | 2 | *Pro-d_5_ | 97.87 ± 7.09 | - | y = 6.31x10^7^ – 7.99x10^7^ | 0.99 | | |  |
|  | 3 | Pro | 98.15 ± 12.43 | 118 | y = 1.27x10^8^ x + 1.63x10^6^ | 0.998 | | |  |
|  | 4 | 2-Mpro | 108.91 ± 10.15 | 120 | y = 3.20x10^8^ x – 5.64x10^6^ | 0.998 | | |  |
|  | 5 | But | 112.57 ± 13.91 | 135 | y = 3.66x10^8^ x – 5.13x10^6^ | 0.997 | | |  |
|  | 6 | 3-Mbut | 112.33 ± 11.49 | 123 | y = 3.66x10^8^ x – 5.43x10^6^ | 0.997 | | |  |
|  | 7 | 2-Mbut | 115.72 ± 10.37 | 125 | y = 2.17x10^8^ x – 5.24x10^6^ | 0.997 | | |  |
|  | 8 | Pen | 117.96 ± 13.08 | 128 | y = 3.05x10^8^ x – 4.74x10^6^ | 0.996 | | |  |
|  | 9 | 3-Mpen | 122.92 ± 13.80 | 128 | y = 2.17x10^8^ x – 3.10x10^6^ | 0.997 | | |  |
|  | 10 | 4-Mpen | 122.82 ± 14.68 | 129 | y = 1.04x10^8^ x – 1.64x10^6^ | 0.995 | | |  |
|  | 11 | Hex | 128.88 ± 16.35 | 135 | y = 1.58x10^8^ x – 4.83x10^5^ | 0.995 | | |  |
|  | 12 | 2-Mhex | 131.42 ± 14.87 | 132 | y = 2.16x10^8^ x – 3.57x10^6^ | 0.996 | | |  |
|  | 13 | 4-Mhex | 131.49 ± 16.91 | 140 | y = 5.92x10^7^ x + 9.85x10^5^ | 0.995 | | |  |
|  | 14 | Hep | 133.74 ± 17.74 | 143 | y = 1.09x10^8^ x – 7.23x10^5^ | 0.997 | | |  |
| *= Internal standard | |  |  |  |  |  | | |  |
| The matrix effect of the quantitative method was examined by comparing the slopes of the calibration curves obtained in the solvent with those obtained from the fecal and plasma matrices. We classified the results into three categories: no matrix effect (80%–120%), moderate matrix effect (40%–80% or 120%–150%), and high matrix effect (<40% or >150%) (1, 2). The plasma samples showed no matrix effects for Pro and 2-Mpro, but showed moderate matrix effects for other SCFAs. In contrast, all SCFAs in the fecal samples showed different levels of matrix effects (Table S3). Thus, quantitative analyses of fecal and plasma SCFAs required matrix-matched calibration curves, similar to those used in a previous study (3).  **References:** 1. Lotti C, Rubert J, Fava F, Tuohy K, Mattivi F, Vrhovsek U. Development of a fast and cost-effective gas chromatography-mass spectrometry method for the quantification of short-chain and medium-chain fatty acids in human biofluids. Anal Bioanal Chem. 2017;409(23):5555-67. 2. Ferrer C, Lozano A, Agüera A, Girón AJ, Fernández-Alba AR. Overcoming matrix effects using the dilution approach in multiresidue methods for fruits and vegetables. J Chromatogr A. 2011;1218(42):7634-9. 3. Limjiasahapong S, Kaewnarin K, Jariyasopit N, Hongthong S, Nuntasaen N, Robinson JL, et al. UPLC-ESI-MRM/MS for Absolute Quantification and MS/MS Structural Elucidation of Six Specialized Pyranonaphthoquinone Metabolites From Ventilago harmandiana. Front Plant Sci. 2020;11:602993. | | | | | | |  |  |  |
|  |  |  |  |  |  |  |  |  |  |
|  |  |  |  |  |  |  |  |  |  |
|  |  |  |  |  |  |  |  |  |  |
|  |  |  |  |  |  |  |  |  |  |
|  |  |  |  |  |  |  |  |  |  |
|  |  |  |  |  |  |  |  |  |  |
|  |  |  |  |  |  |  |  |  |  |
|  |  |  |  |  |  |  |  |  |  |
|  |  |  |  |  |  |  |  |  |  |
|  |  |  |  |  |  |  |  |  |  |
|  |  |  |  |  |  |  |  |  |  |
|  |  |  |  |  |  |  |  |  |  |
|  |  |  |  |  |  |  |  |  |  |
|  |  |  |  |  |  |  |  |  |  |
|  |  |  |  |  |  |  |  |  |  |

| **Supplementary Table S4. List of SCFAs and GC-MS characteristics of SCFAs in fecal and plasma matrix** | | | | | | | | |  |  |  |  |
| --- | --- | --- | --- | --- | --- | --- | --- | --- | --- | --- | --- | --- |
| **Sample** |  | **Standards** | **RT (mins)** | **LOD  (ng/mL)** | **LOQ (ng/mL)** | **%RSD of Intraday (N=10)** | | **%RSD of Interday (N=3)** | | **Calibration Curve** | **R^2^** | **Linear Range** |
|  |  |  |  | **(N=10)** | **(N=10)** | **Peak area** | **RT** | **Peak area** | **RT** |  |  | **(ng/µL)** |
| **Fecal Sample** | 1 | Ace | 6.39 | 50 | 152 | 1.89 | 0.017 | 5.78 | 0.003 | y = 1.741x + 9.897 | 0.999 | 0.050 - 70 |
|  | 2 | *Pro-d5 | 7.04 | 27 | 82 | 2.07 | 0.011 | 6.52 | 0.003 | NA | NA | 0.050 - 70 |
|  | 3 | Pro | 7.1 | 23 | 69 | 2.06 | 0.278 | 6.46 | 0.003 | y = 0.648x + 3.762 | 0.999 | 0.025 - 70 |
|  | 4 | 2-Mpro | 7.33 | 21 | 65 | 1.6 | 0.011 | 5.71 | 0.002 | y = 3.627x + 1.949 | 0.999 | 0.025 - 70 |
|  | 5 | But | 7.82 | 9 | 28 | 1.61 | 0.009 | 9.5 | 0.006 | y = 2.346x + 12.103 | 0.999 | 0.013 - 70 |
|  | 6 | 3-Mbut | 8.13 | 8 | 23 | 2.21 | 0.007 | 8.2 | 0.004 | y = 3.597x + 1.628 | 0.999 | 0.013 - 70 |
|  | 7 | 2-Mbut | 8.14 | 48 | 147 | 1.68 | 0.007 | 6.74 | 0.003 | y = 1.079x + 0.717 | 0.999 | 0.500 - 50 |
|  | 8 | Pen | 8.66 | 12 | 36 | 1.65 | 0.007 | 7.83 | 0.004 | y = 4.107x + 2.368 | 0.999 | 0.025 - 70 |
|  | 9 | 3-Mpen | 9.06 | 11 | 35 | 1.49 | 0.005 | 7.88 | 0.004 | y = 2.180x + 0.197 | 0.999 | 0.025 - 70 |
|  | 10 | 4-Mpen | 9.14 | 13 | 41 | 1.89 | 0.22 | 8.19 | 0.002 | y = 1.722x + 0.095 | 0.999 | 0.025 - 50 |
|  | 11 | Hex | 9.44 | 16 | 48 | 3.34 | 0.003 | 6.46 | 0.003 | y = 2.502x + 0.289 | 0.999 | 0.025 - 70 |
|  | 12 | 2-Mhex | 9.6 | 16 | 48 | 1.66 | 0.004 | 8.5 | 0.002 | y = 2.519x – 0.054 | 0.999 | 0.025 - 60 |
|  | 13 | 4-Mhex | 10.01 | 7 | 22 | 2.22 | 0.004 | 9.82 | 0.002 | y = 1.095x + 0.091 | 0.999 | 0.025 - 50 |
|  | 14 | Hep | 10.18 | 5 | 14 | 2.63 | 0.004 | 11.1 | 0.002 | y = 3.385x + 0.152 | 0.999 | 0.006 - 70 |
| **Sample** |  | **Standards** | **RT (mins)** | **LOD (µM)** | **LOQ (µM)** | **%RSD of Intraday (N=10)** | | **%RSD of Interday (N=3)** | | **Calibration Curve** | **R^2^** | **Linear Range** |
|  |  |  |  | **(N=10)** | **(N=10)** | **Peak area** | **RT** | **Peak area** | **RT** |  |  | **(µM)** |
| **Plasma Sample** | 1 | Ace | 6.34 | 5.14 | 17.133 | 5.38 | 0.004 | 9.18 | 0.004 | y = 0.590x + 0.530 | 0.994 | 5.204 - 42 |
|  | 2 | *Pro-d_5_ | 6.98 | 0.161 | 0.538 | 0 | 0.006 | 10.69 | 0.005 | NA | NA | 0.247 - 32 |
|  | 3 | Pro | 7.04 | 0.245 | 0.816 | 4.24 | 0.285 | 1.89 | 0.472 | y = 0.332x + 0.042 | 0.998 | 0.264 - 34 |
|  | 4 | 2-Mpro | 7.27 | 0.035 | 0.115 | 4.02 | 0.006 | 2.61 | 0.002 | y = 0.911x + 0.055 | 0.998 | 0.055 - 28 |
|  | 5 | But | 7.76 | 0.015 | 0.051 | 4.47 | 0.005 | 4.96 | 0.002 | y = 0.981x + 0.056 | 0.997 | 0.028 - 28 |
|  | 6 | 3-Mbut | 8.06 | 0.01 | 0.034 | 4.31 | 0.005 | 5.62 | 0.002 | y = 0.992x + 0.044 | 0.995 | 0.024 - 24 |
|  | 7 | 2-Mbut | 8.08 | 0.074 | 0.248 | 3.86 | 0.005 | 4.43 | 0.001 | y = 0.557x + 0.027 | 0.997 | 0.096 - 24 |
|  | 8 | Pen | 8.6 | 0.011 | 0.035 | 4.67 | 0.004 | 9.37 | 0.002 | y = 0.857x + 0.031 | 0.995 | 0.024 - 24 |
|  | 9 | 3-Mpen | 9 | 0.007 | 0.024 | 4.32 | 0.003 | 9.76 | 0.001 | y = 0.608x + 0.023 | 0.996 | 0.021 - 22 |
|  | 10 | 4-Mpen | 9.07 | 0.018 | 0.059 | 4.6 | 0.005 | 11.06 | 0.002 | y =0.306x + 0.010 | 0.991 | 0.021 - 22 |
|  | 11 | Hex | 9.38 | 0.015 | 0.051 | 4.99 | 0.212 | 13.21 | 0.002 | y = 0.465x + 0.023 | 0.993 | 0.021 - 22 |
|  | 12 | 2-Mhex | 9.53 | 0.038 | 0.127 | 4.35 | 0.004 | 12.76 | 0.002 | y = 0.594x + 0.018 | 0.994 | 0.075 - 19 |
|  | 13 | 4-Mhex | 9.94 | 0.032 | 0.107 | 4.9 | 0.003 | 14.47 | 0.001 | y = 0.179x + 0.010 | 0.992 | 0.075 - 19 |
|  | 14 | Hep | 10.12 | 0.057 | 0.171 | 5.97 | 0.004 | 17.35 | 0.001 | y = 0.336x + 0.015 | 0.99 | 0.019 - 19 |
| *= Internal standard |  |  |  |  |  |  |  |  |  |  |  |  |

| **Supplementary Table S5. Median amount of consumption for some macronutrients** | | | |  |  |
| --- | --- | --- | --- | --- | --- |
|  | **Median ± MAD** | | | | |
| **Nutrients** | **Females (N=98)** | Daily Thai female DRIs | **Males (N=59)** | Daily Thai male DRIs | **Overall (N=157)** |
| ***Energy (kcal)** | 1610.7 ± 282.6 | 1500 - 1800 | 1732.4 ± 309.6 | 1800 - 2200 | 1655.1 ± 278.4 |
| ***Carbohydrate (g)** | 173.9 ± 29.4 | 242 - 414 | 199.7 ± 34.0 | 298 - 525 | 182.7 ± 32.3 |
| ***Protein (g)** | 70.1 ± 20.0 | 51.9 - 53.0 | 81.5 ± 16.4 | 59.5 -61.3 | 76.5 ± 18.25 |
| **Protein from animals(g)** | 46.65 ± 20.0 |  | 54.8 ± 16.1 |  | 47.4 ± 18.2 |
| ***Protein from vegetables (g)** | 10.4 ± 2.4 |  | 13.2 ± 2.7 |  | 12.1 ± 3.3 |
| **Fat (g)** | 59.8 ± 85.4 | 45 - 78 | 65.7 ± 14.5 | 45 - 78 | 61.35 ± 13.8 |
| **Vitamin B1 (mg)** | 0.9 ± 0.4 | > 1.1 | 1.2 ± 0.5 | > 1.2 | 1.1 ± 0.5 |
| ******Vitamin B2 (mg)** | 1.0 ± 0.3 | > 1.1 | 1.5 ± 0.3 | > 1.3 | 1.25 ± 0.35 |
| *****Vitamin B3 (Niacin, mg)** | 12.9 ± 4.35 | > 14 | 17.4 ± 5.2 | > 16 | 14.30 ± 4.65 |
| ***Vitamin C (mg)** | 41.2 ± 24 | > 85 | 25.3 ± 16.3 | > 100 | 30.6 ± 19.7 |
| *****Vitamin A (Retinol, µg)** | 244.1 ± 108.0 | > 600 | 338.6 ± 142.6 | > 700 | 276.1 ± 125.85 |
| ******Calcium (mg)** | 360.2 ± 95.2 | 800-1000 | 532.7 ± 144.7 | 800-1000 | 422.9 ± 136.3 |
| **Iron (mg)** | 9.3 ± 2.6 | 10.00-20.00 | 9.5 ± 2.8 | 11.0-11.5 | 9.3 ± 2.7 |
| **Iron from animals (mg)** | 3.3 ± 1.4 | 10.4 | 4.1 ± 1.6 | 24.7 | 3.6 ± 1.5 |
| **Iron from vegetables (mg)** | 3.2 ± 3.2 |  | 3.3 ± 3.3 |  | 3.2 ± 3.2 |
| **Crude Fiber (g)** | 0.1 ± 0.1 | 25 | 0.2 ± 0.2 | 25 | 0.2 ± 0.2 |
| **Dietary Fiber (g)** | 7.9 ± 7.7 |  | 7.3 ± 7.2 |  | 7.6 ± 2.8 |
| Analysed by Mann-Whitney U unpaired T-test | |  |  |  |  |
| * Statistically significant between genders (p-value < 0.05) | | |  |  |  |
| *** Statistically significant between genders (p-value < 0.005) | | |  |  |  |
| **** Statistically significant between genders (p-value < 0.0001) | | |  |  |  |

| **Supplementary Table S6. SCFAs Concentration in Feces sample (N=157)** | | | | | | | | | | | | | |
| --- | --- | --- | --- | --- | --- | --- | --- | --- | --- | --- | --- | --- | --- |
| **Subjects** | **Concentration (µmol/g)** | | | | | | | | | | | | |
|  | **Ace** | **Pro** | **2-Mpro** | **But** | **3-Mbut** | **2-Mbut** | **Pen** | **3-Mpen** | **4-Mpen** | **Hex** | **2-Mhex** | **4-Mhex** | **Hep** |
| **1_G1_C1_003** | 11.71 | 9.65 | 0.75 | 5.69 | 0.64 | 1.08 | 0.95 | <LOQ | <LOQ | <LOQ | 0.0088 | <LOQ | <LOQ |
| **2_G1_C1_006** | 9.94 | 11.12 | 0.66 | 1.92 | 0.48 | 0.74 | 0.70 | <LOQ | <LOQ | <LOQ | 0.0063 | <LOQ | <LOQ |
| **3_G1_C1_008** | 12.31 | 10.77 | <LOQ | 15.56 | <LOQ | <LOQ | 0.56 | <LOQ | 0.02 | <LOQ | 0.0095 | <LOQ | <LOQ |
| **4_G1_C1_009** | 4.14 | 1.35 | 0.36 | 0.18 | 0.23 | 0.51 | 0.31 | <LOQ | 0.07 | 0.01 | 0.0063 | <LOQ | <LOQ |
| **5_G1_C1_010** | 8.22 | 7.13 | 0.07 | 4.15 | 0.05 | 0.02 | 0.32 | <LOQ | <LOQ | <LOQ | 0.0061 | <LOQ | <LOQ |
| **6_G1_C1_011** | 13.22 | 8.04 | 0.66 | 9.40 | 0.58 | 0.87 | 1.10 | <LOQ | 0.01 | 0.18 | 0.0068 | <LOQ | <LOQ |
| **7_G1_C1_012** | 10.75 | 6.03 | <LOQ | 7.12 | <LOQ | <LOQ | 0.20 | <LOQ | <LOQ | <LOQ | 0.0065 | <LOQ | <LOQ |
| **8_G1_C1_014** | 8.18 | 8.03 | 0.37 | 4.07 | 0.25 | 0.42 | 0.42 | <LOQ | 0.02 | <LOQ | 0.0115 | <LOQ | <LOQ |
| **9_G1_C1_015** | 12.61 | 9.57 | 0.51 | 7.83 | 0.40 | 0.41 | 1.15 | <LOQ | <LOQ | 0.72 | 0.0064 | <LOQ | 0.08 |
| **10_G1_C1_018** | 14.50 | 16.41 | 0.29 | 9.27 | 0.24 | 0.35 | 0.94 | <LOQ | 0.02 | <LOQ | 0.0098 | <LOQ | <LOQ |
| **11_G1_C2_001** | 0.53 | 1.20 | 0.38 | 2.48 | 0.44 | 0.61 | 0.43 | <LOQ | <LOQ | <LOQ | 0.0062 | <LOQ | <LOQ |
| **12_G1_C2_003** | 9.31 | 1.69 | 0.03 | 17.62 | 0.04 | 0.01 | 0.46 | <LOQ | <LOQ | 0.16 | 0.0060 | <LOQ | <LOQ |
| **13_G1_C2_004** | 12.63 | 8.89 | 0.98 | 8.20 | 0.95 | 1.16 | 1.55 | <LOQ | <LOQ | 0.69 | 0.0109 | <LOQ | 0.11 |
| **14_G1_C2_005** | 8.46 | 4.55 | 0.13 | 2.84 | 0.11 | 0.13 | ND | <LOQ | <LOQ | <LOQ | 0.0077 | <LOQ | <LOQ |
| **15_G1_C2_006** | 1.91 | 0.61 | 0.22 | 0.35 | 0.25 | 0.35 | 0.11 | <LOQ | <LOQ | <LOQ | 0.0066 | <LOQ | <LOQ |
| **16_G1_C2_007** | 10.41 | 5.23 | 0.49 | 8.52 | 0.41 | 0.59 | 0.64 | <LOQ | 0.02 | <LOQ | 0.0060 | <LOQ | <LOQ |
| **17_G1_C2_008** | 7.88 | 9.61 | 0.77 | 3.90 | 0.68 | 0.99 | 0.64 | <LOQ | <LOQ | <LOQ | 0.0064 | <LOQ | <LOQ |
| **18_G1_C2_009** | 10.38 | 8.04 | 0.17 | 9.31 | 0.15 | 0.19 | 0.89 | <LOQ | <LOQ | <LOQ | 0.0075 | <LOQ | <LOQ |
| **19_G1_C2_010** | 11.50 | 9.91 | 0.67 | 10.08 | 0.56 | 0.93 | 0.89 | <LOQ | <LOQ | <LOQ | 0.0062 | <LOQ | <LOQ |
| **20_G1_C2_011** | 6.29 | 6.68 | 0.50 | 7.07 | 0.46 | 0.74 | 0.83 | <LOQ | <LOQ | <LOQ | 0.0062 | <LOQ | <LOQ |
| **21_G2_C1_001** | 1.48 | 5.60 | 0.87 | 4.89 | 0.77 | 1.19 | 0.79 | <LOQ | 0.01 | 0.01 | 0.0113 | <LOQ | 0.01 |
| **22_G2_C1_003** | 11.86 | 8.15 | <LOQ | 4.19 | <LOQ | <LOQ | 0.15 | <LOQ | <LOQ | 0.11 | 0.0063 | <LOQ | 0.01 |
| **23_G2_C1_004** | 12.22 | 9.95 | 0.70 | 6.05 | 0.67 | 0.98 | 0.92 | <LOQ | <LOQ | 0.26 | 0.0064 | <LOQ | 0.03 |
| **24_G2_C1_005** | 16.10 | 15.13 | 0.40 | 19.41 | 0.33 | 0.48 | 2.07 | <LOQ | <LOQ | 0.22 | 0.0110 | <LOQ | <LOQ |
| **25_G2_C1_006** | 22.57 | 25.54 | 0.59 | 25.07 | 0.48 | 0.72 | 1.81 | <LOQ | 0.05 | 0.02 | 0.0067 | <LOQ | <LOQ |
| **26_G2_C1_007** | 9.86 | 8.85 | 0.17 | 2.39 | 0.11 | 0.15 | 0.50 | <LOQ | 0.01 | <LOQ | 0.0074 | <LOQ | <LOQ |
| **27_G2_C1_009** | 15.12 | 10.69 | 0.76 | 13.56 | 0.62 | 0.94 | 0.89 | <LOQ | <LOQ | 0.01 | 0.0060 | <LOQ | <LOQ |
| **28_G2_C1_010** | 11.31 | 10.54 | 0.80 | 9.54 | 0.68 | 0.90 | 0.94 | <LOQ | 0.02 | <LOQ | 0.0081 | <LOQ | <LOQ |
| **29_G2_C1_012** | 8.52 | 4.30 | 1.27 | 7.27 | 1.36 | 2.13 | 1.27 | <LOQ | <LOQ | 0.68 | 0.0119 | <LOQ | 0.10 |
| **30_G2_C1_013** | 12.05 | 17.62 | <LOQ | 8.78 | <LOQ | <LOQ | 0.12 | <LOQ | <LOQ | <LOQ | 0.0060 | <LOQ | <LOQ |
| **31_G2_C1_014** | 10.72 | 11.48 | 1.07 | 9.86 | 0.94 | 1.61 | 1.40 | <LOQ | 0.02 | 0.02 | 0.0094 | <LOQ | <LOQ |
| **32_G2_C1_015** | 8.05 | 7.50 | 0.16 | 5.15 | 0.12 | 0.16 | 1.22 | <LOQ | <LOQ | <LOQ | 0.0071 | <LOQ | <LOQ |
| **33_G2_C1_017** | 10.60 | 5.41 | 0.32 | 7.76 | 0.30 | 0.47 | ND | <LOQ | <LOQ | <LOQ | 0.0061 | <LOQ | <LOQ |
| **34_G2_C2_001** | 16.08 | 11.84 | 0.31 | 12.93 | 0.20 | 0.25 | 0.93 | <LOQ | 0.02 | <LOQ | 0.0096 | <LOQ | <LOQ |
| **35_G2_C2_002** | 8.45 | 7.95 | 0.59 | 8.24 | 0.51 | 0.82 | 0.58 | <LOQ | 0.01 | <LOQ | 0.0074 | <LOQ | <LOQ |
| **36_G2_C2_003** | 5.57 | 6.42 | 0.62 | 3.15 | 0.58 | 0.93 | ND | <LOQ | 0.01 | <LOQ | 0.0061 | <LOQ | <LOQ |
| **37_G2_C2_005** | 5.11 | 3.30 | 0.52 | 2.86 | 0.50 | 0.83 | 0.75 | <LOQ | 0.01 | 0.54 | 0.0126 | <LOQ | <LOQ |
| **38_G2_C2_006** | 9.28 | 7.60 | 1.07 | 4.61 | 0.90 | 1.33 | 2.00 | <LOQ | <LOQ | 0.16 | 0.0099 | <LOQ | <LOQ |
| **39_G2_C2_007** | 8.76 | 7.02 | 0.22 | 5.26 | 0.22 | 0.29 | 0.23 | <LOQ | <LOQ | <LOQ | 0.0059 | <LOQ | <LOQ |
| **40_G2_C2_008** | 4.02 | 5.35 | 0.31 | 2.02 | 0.36 | 0.57 | 0.43 | <LOQ | 0.01 | <LOQ | 0.0060 | <LOQ | <LOQ |
| **41_G3_C1_001** | 17.03 | 16.03 | 0.42 | 17.80 | 0.59 | 0.96 | 1.03 | <LOQ | 0.32 | <LOQ | 0.0079 | <LOQ | <LOQ |
| **42_G3_C1_002** | 15.42 | 20.04 | 0.15 | 13.58 | 0.11 | 0.08 | 1.17 | <LOQ | 0.02 | 0.08 | 0.0055 | <LOQ | <LOQ |
| **43_G3_C1_003** | 13.79 | 11.15 | 2.13 | 16.28 | 2.09 | 3.35 | 1.69 | <LOQ | 0.02 | 0.03 | 0.0095 | <LOQ | <LOQ |
| **44_G3_C1_004** | 10.40 | 4.70 | 0.13 | 4.37 | 0.10 | 0.09 | 0.19 | <LOQ | <LOQ | <LOQ | 0.0055 | <LOQ | <LOQ |
| **45_G3_C1_005** | 9.85 | 7.75 | 0.89 | 8.56 | 0.76 | 1.05 | 1.60 | <LOQ | <LOQ | 0.06 | 0.0068 | <LOQ | <LOQ |
| **46_G3_C1_010** | 4.85 | 3.36 | 0.22 | 4.41 | 0.21 | 0.30 | 0.56 | <LOQ | <LOQ | 0.04 | 0.0089 | <LOQ | <LOQ |
| **47_G3_C1_011** | 12.61 | 9.80 | 0.62 | 7.41 | 0.55 | 0.83 | 1.05 | <LOQ | 0.01 | 0.17 | 0.0055 | <LOQ | <LOQ |
| **48_G3_C1_012** | 12.10 | 10.81 | 0.70 | 10.72 | 0.49 | 0.84 | 0.79 | <LOQ | 0.02 | <LOQ | 0.0066 | <LOQ | <LOQ |
| **49_G3_C1_013** | 17.02 | 22.83 | 1.25 | 18.85 | 1.26 | 1.80 | 2.35 | <LOQ | <LOQ | 0.35 | 0.0099 | <LOQ | 0.02 |
| **50_G3_C1_014** | 19.39 | 17.69 | 0.95 | 11.87 | 0.84 | 1.49 | 1.98 | <LOQ | 0.01 | 0.08 | 0.0060 | <LOQ | <LOQ |
| **51_G3_C1_015** | 2.90 | 2.15 | 0.34 | 2.79 | 0.33 | 0.52 | 0.36 | <LOQ | <LOQ | 0.36 | 0.0069 | <LOQ | 0.05 |
| **52_G3_C1_016** | 20.51 | 19.18 | 0.88 | 6.80 | 0.68 | 1.31 | 2.64 | <LOQ | <LOQ | 0.29 | 0.0061 | <LOQ | <LOQ |
| **53_G3_C1_017** | 12.18 | 12.41 | 0.46 | 6.60 | 0.40 | 0.44 | ND | <LOQ | 0.01 | <LOQ | 0.0066 | <LOQ | <LOQ |
| **54_G3_C2_001** | 5.85 | 7.59 | 1.14 | 9.49 | 0.40 | 1.80 | 1.21 | <LOQ | 0.02 | 0.01 | 0.0063 | <LOQ | <LOQ |
| **55_G3_C2_002** | 4.92 | 3.98 | 0.73 | 5.88 | 0.65 | 1.04 | 1.01 | <LOQ | ND | 0.08 | 0.0071 | <LOQ | <LOQ |
| **56_G3_C2_003** | 13.77 | 13.53 | 0.59 | 9.31 | 0.52 | 0.49 | 1.12 | <LOQ | 0.01 | 0.02 | 0.0089 | <LOQ | <LOQ |
| **57_G3_C2_004** | 10.51 | 6.69 | ND | 12.15 | ND | ND | ND | <LOQ | <LOQ | <LOQ | 0.0081 | <LOQ | <LOQ |
| **58_G3_C2_005** | 14.15 | 10.65 | 0.23 | 6.61 | 0.16 | 0.18 | 1.40 | <LOQ | <LOQ | 0.03 | 0.0067 | <LOQ | <LOQ |
| **59_G3_C2_006** | 11.23 | 10.48 | 0.38 | 9.84 | 0.30 | 0.45 | 0.72 | <LOQ | <LOQ | <LOQ | 0.0082 | <LOQ | 0.01 |
| **60_G3_C2_007** | 10.63 | 13.09 | 0.77 | 0.57 | 0.62 | 0.93 | 0.85 | <LOQ | 0.01 | <LOQ | 0.0062 | <LOQ | <LOQ |
| **61_H_0001** | 18.71 | 7.09 | 2.23 | 6.71 | 1.96 | 1.29 | 3.59 | <LOQ | <LOQ | 0.46 | <LOQ | <LOQ | <LOQ |
| **62_H_0002** | 29.62 | 26.66 | 4.33 | 16.56 | 3.09 | 2.51 | 4.77 | <LOQ | 0.04 | 0.06 | <LOQ | <LOQ | <LOQ |
| **63_H_0003** | 18.97 | 16.17 | 0.78 | 10.38 | 0.58 | 0.38 | 1.62 | <LOQ | 0.09 | <LOQ | <LOQ | <LOQ | <LOQ |
| **64_H_0004** | 13.41 | 6.44 | 2.10 | 7.07 | 1.66 | 1.29 | 2.30 | <LOQ | 0.03 | 0.05 | <LOQ | <LOQ | <LOQ |
| **65_H_0005** | 28.36 | 15.33 | 1.69 | 18.66 | 1.34 | 0.95 | 3.54 | <LOQ | 0.05 | 0.08 | <LOQ | <LOQ | <LOQ |
| **66_H_0006** | 62.42 | 50.00 | 3.33 | 43.09 | 2.93 | 2.10 | 5.55 | <LOQ | 0.10 | 0.12 | <LOQ | <LOQ | 0.02 |
| **67_H_0007** | 7.27 | 3.63 | 0.75 | 4.82 | 0.46 | 0.36 | 1.04 | <LOQ | <LOQ | <LOQ | <LOQ | <LOQ | <LOQ |
| **68_H_0008** | 18.36 | 10.95 | 0.51 | 5.13 | 0.40 | 0.18 | 1.11 | <LOQ | <LOQ | <LOQ | <LOQ | <LOQ | <LOQ |
| **69_H_0009** | 24.86 | 11.02 | 1.41 | 13.88 | 0.83 | 0.62 | 2.28 | <LOQ | 0.05 | <LOQ | <LOQ | <LOQ | <LOQ |
| **70_H_0010** | 6.32 | 6.61 | 2.32 | 5.67 | 1.95 | 1.70 | 1.46 | <LOQ | <LOQ | 0.04 | <LOQ | <LOQ | <LOQ |
| **71_H_0011** | 24.81 | 6.83 | 0.16 | 7.29 | 0.02 | ND | 0.08 | <LOQ | <LOQ | <LOQ | <LOQ | <LOQ | <LOQ |
| **72_H_0012** | 30.11 | 14.81 | 0.75 | 12.09 | 0.46 | 0.38 | 1.96 | <LOQ | 0.07 | 0.05 | <LOQ | <LOQ | <LOQ |
| **73_H_0013** | 23.72 | 16.59 | 3.01 | 11.58 | 2.59 | 1.66 | 2.00 | <LOQ | 0.13 | 0.06 | <LOQ | <LOQ | <LOQ |
| **74_H_0014** | 34.78 | 17.32 | 3.00 | 15.68 | 2.00 | 1.85 | 4.56 | <LOQ | 0.42 | 0.10 | <LOQ | <LOQ | 0.03 |
| **75_H_0015** | 5.93 | 4.98 | 0.85 | 0.53 | 0.52 | 0.41 | 0.89 | <LOQ | 0.03 | <LOQ | <LOQ | <LOQ | <LOQ |
| **76_H_0016** | 6.41 | 3.94 | 1.70 | 0.77 | 1.41 | 1.07 | 1.74 | <LOQ | 0.04 | 0.04 | <LOQ | <LOQ | <LOQ |
| **77_H_0017** | 40.30 | 24.99 | 4.37 | 17.24 | 2.98 | 1.98 | 4.67 | <LOQ | 0.07 | <LOQ | <LOQ | <LOQ | <LOQ |
| **78_H_0018** | 40.23 | 13.90 | 0.34 | 27.93 | 0.23 | 0.13 | 1.13 | <LOQ | 0.05 | 0.05 | <LOQ | <LOQ | <LOQ |
| **80_H_0021** | 31.32 | 28.13 | 2.46 | 15.90 | 1.40 | 1.31 | 6.39 | <LOQ | 0.09 | 0.06 | <LOQ | <LOQ | <LOQ |
| **81_H_0022** | 30.21 | 19.29 | 1.02 | 20.06 | 0.55 | 0.41 | 2.45 | <LOQ | 0.06 | <LOQ | <LOQ | <LOQ | <LOQ |
| **82_H_0023** | 0.57 | 0.77 | 1.32 | 1.96 | 0.93 | 0.71 | 1.07 | <LOQ | <LOQ | 0.09 | <LOQ | <LOQ | <LOQ |
| **83_H_0024** | 8.36 | 7.39 | 4.71 | 9.69 | 4.80 | 3.27 | 5.85 | <LOQ | <LOQ | 0.57 | <LOQ | <LOQ | <LOQ |
| **84_H_0025** | 23.66 | 15.06 | 2.07 | 15.54 | 1.69 | 1.19 | 4.03 | <LOQ | 0.07 | 1.04 | <LOQ | <LOQ | 0.17 |
| **85_H_0026** | 1.29 | 0.23 | 0.99 | 0.11 | 0.69 | 0.55 | 0.65 | <LOQ | <LOQ | 0.02 | <LOQ | <LOQ | <LOQ |
| **86_H_0027** | 12.64 | 4.05 | 0.64 | 12.23 | 0.41 | 0.35 | 1.32 | <LOQ | 0.04 | 0.03 | <LOQ | <LOQ | <LOQ |
| **87_H_0028** | 6.14 | 2.68 | 1.55 | 3.80 | 1.21 | 0.98 | 1.69 | <LOQ | <LOQ | 0.05 | <LOQ | <LOQ | <LOQ |
| **88_H_0029** | 16.50 | 8.48 | 0.94 | 6.75 | 0.56 | 0.35 | 2.50 | <LOQ | 0.04 | <LOQ | <LOQ | <LOQ | <LOQ |
| **89_H_0030** | 32.78 | 18.25 | 1.35 | 5.05 | 0.87 | 0.58 | 1.85 | <LOQ | 0.07 | <LOQ | <LOQ | <LOQ | <LOQ |
| **90_H_0031** | 3.31 | 2.75 | 1.00 | 3.48 | 0.97 | 0.81 | 1.27 | <LOQ | 0.03 | 0.04 | <LOQ | <LOQ | <LOQ |
| **91_H_0032** | 14.15 | 10.88 | 1.43 | 3.70 | 1.11 | 0.87 | 2.21 | <LOQ | <LOQ | 0.06 | <LOQ | <LOQ | <LOQ |
| **92_H_0033** | 36.99 | 21.67 | 1.16 | 18.73 | 0.83 | 0.45 | 3.25 | <LOQ | <LOQ | ND | <LOQ | <LOQ | <LOQ |
| **93_H_0034** | 16.28 | 10.92 | 2.10 | 10.73 | 1.64 | 1.24 | 2.52 | <LOQ | <LOQ | 0.03 | <LOQ | <LOQ | <LOQ |
| **94_H_0035** | 10.46 | 5.22 | 0.80 | 7.23 | 0.56 | 0.41 | 1.54 | <LOQ | 0.03 | <LOQ | <LOQ | <LOQ | <LOQ |
| **95_H_0036** | 6.17 | 3.05 | 0.59 | 4.33 | 0.34 | 0.30 | 0.80 | <LOQ | <LOQ | <LOQ | <LOQ | <LOQ | <LOQ |
| **96_H_0037** | 12.66 | 10.03 | 1.48 | 8.60 | 0.95 | 0.77 | 1.54 | <LOQ | 0.05 | <LOQ | <LOQ | <LOQ | <LOQ |
| **97_H_0038** | 39.42 | 24.77 | 3.47 | 20.66 | 2.40 | 1.95 | 5.34 | <LOQ | 0.09 | 0.06 | <LOQ | <LOQ | <LOQ |
| **98_H_0039** | 29.42 | 20.37 | 3.07 | 12.11 | 2.55 | 1.73 | 4.11 | <LOQ | 0.06 | 0.08 | <LOQ | <LOQ | <LOQ |
| **99_H_0040** | 5.83 | 1.96 | 2.41 | 5.24 | 2.17 | 1.71 | 2.18 | <LOQ | <LOQ | 0.22 | <LOQ | <LOQ | 0.04 |
| **100_H_0041** | 13.99 | 14.25 | 2.53 | 15.95 | 1.79 | 1.53 | 2.98 | <LOQ | 0.03 | 0.07 | <LOQ | <LOQ | <LOQ |
| **101_H_0042** | 5.10 | 2.44 | 0.73 | 6.43 | 0.49 | 0.38 | 0.91 | <LOQ | <LOQ | 0.04 | <LOQ | <LOQ | <LOQ |
| **102_H_0043** | 17.78 | 14.37 | 2.04 | 5.41 | 1.61 | 1.29 | 2.71 | <LOQ | <LOQ | 0.15 | <LOQ | <LOQ | <LOQ |
| **103_H_0044** | 13.42 | 8.06 | 0.73 | 5.49 | 0.50 | 0.33 | 1.33 | <LOQ | 0.05 | <LOQ | <LOQ | <LOQ | <LOQ |
| **104_H_0045** | 7.33 | 3.22 | 0.79 | 2.43 | 0.61 | 0.44 | 1.02 | <LOQ | <LOQ | <LOQ | <LOQ | <LOQ | <LOQ |
| **105_H_0046** | 36.06 | 19.26 | 0.21 | 20.20 | 0.20 | ND | 0.49 | <LOQ | 0.05 | <LOQ | <LOQ | <LOQ | <LOQ |
| **106_H_0047** | 29.96 | 17.09 | 2.19 | 18.44 | 1.60 | 1.19 | 5.24 | <LOQ | 0.10 | 0.07 | <LOQ | <LOQ | <LOQ |
| **107_H_0048** | 11.03 | 5.71 | 2.23 | 6.07 | 1.72 | 1.64 | 2.60 | <LOQ | 0.06 | 1.80 | <LOQ | <LOQ | 0.43 |
| **108_H_0049** | 15.89 | 13.17 | 2.05 | 9.26 | 1.58 | 1.20 | 2.83 | <LOQ | <LOQ | 0.06 | <LOQ | <LOQ | <LOQ |
| **109_H_0050** | 39.69 | 20.02 | 2.07 | 11.01 | 1.25 | 0.98 | 4.38 | <LOQ | 0.05 | <LOQ | <LOQ | <LOQ | <LOQ |
| **110_H_0051** | 13.80 | 10.49 | 0.89 | 4.41 | 0.56 | 0.41 | 2.06 | <LOQ | 0.05 | <LOQ | <LOQ | <LOQ | <LOQ |
| **111_H_0052** | 0.18 | 12.67 | 2.52 | 5.55 | 2.02 | 1.61 | 2.58 | <LOQ | 0.03 | 0.07 | <LOQ | <LOQ | <LOQ |
| **112_H_0053** | 9.77 | 8.92 | 1.31 | 5.73 | 1.02 | 0.65 | 1.92 | <LOQ | <LOQ | <LOQ | <LOQ | <LOQ | <LOQ |
| **113_H_0054** | 26.78 | 24.84 | 1.89 | 11.99 | 1.39 | 1.10 | 4.11 | <LOQ | <LOQ | 0.04 | <LOQ | <LOQ | <LOQ |
| **114_H_0055** | 2.78 | 1.35 | 0.76 | 1.76 | 0.55 | 0.46 | 1.38 | <LOQ | <LOQ | 0.09 | <LOQ | <LOQ | <LOQ |
| **115_H_0056** | 19.78 | 18.44 | 1.87 | 1.12 | 1.27 | 0.85 | 0.03 | <LOQ | <LOQ | <LOQ | <LOQ | <LOQ | <LOQ |
| **116_H_0057** | ND | ND | 0.78 | 0.44 | 0.59 | 0.50 | 0.70 | <LOQ | <LOQ | 0.28 | <LOQ | <LOQ | 0.06 |
| **117_H_0058** | 3.41 | 2.99 | 0.93 | ND | 0.69 | 0.55 | 0.04 | <LOQ | 0.02 | <LOQ | <LOQ | <LOQ | <LOQ |
| **118_H_0059** | 1.23 | 0.28 | 1.87 | 1.18 | 1.53 | 1.30 | 1.16 | <LOQ | <LOQ | 0.14 | <LOQ | <LOQ | 0.02 |
| **119_H_0060** | 3.36 | 3.46 | 2.17 | 2.70 | 1.88 | 1.58 | 4.14 | <LOQ | <LOQ | 0.40 | <LOQ | <LOQ | <LOQ |
| **120_H_0061** | 17.76 | 6.45 | 0.68 | 12.80 | 0.46 | 0.33 | 2.14 | <LOQ | <LOQ | 0.04 | <LOQ | <LOQ | <LOQ |
| **121_H_0062** | 11.42 | 8.30 | 1.90 | 4.08 | 1.53 | 1.38 | 2.31 | <LOQ | <LOQ | 0.03 | <LOQ | <LOQ | <LOQ |
| **122_H_0064** | 15.76 | 17.61 | 0.64 | 5.57 | 0.42 | 0.29 | 1.89 | <LOQ | <LOQ | <LOQ | <LOQ | <LOQ | <LOQ |
| **123_H_0065** | 28.41 | 14.87 | 1.58 | 23.39 | 1.44 | 1.07 | 4.31 | <LOQ | 0.13 | 0.25 | <LOQ | <LOQ | <LOQ |
| **124_H_0067** | 7.42 | 2.64 | 0.63 | 3.65 | 0.34 | 0.31 | 1.41 | <LOQ | <LOQ | 0.17 | <LOQ | <LOQ | <LOQ |
| **125_H_0068** | 2.24 | <LOQ | 1.24 | <LOQ | 0.94 | 0.75 | 1.07 | <LOQ | <LOQ | 0.14 | <LOQ | <LOQ | 0.05 |
| **126_H_0069** | 12.32 | 5.20 | 1.03 | <LOQ | 0.76 | 0.69 | 0.23 | <LOQ | 0.03 | <LOQ | <LOQ | <LOQ | <LOQ |
| **127_H_0070** | 3.87 | 4.48 | 0.14 | <LOQ | 0.07 | 0.02 | 0.08 | <LOQ | 0.02 | <LOQ | <LOQ | <LOQ | <LOQ |
| **128_H_0071** | 28.53 | 19.94 | 1.38 | 15.60 | 1.04 | 0.65 | 4.01 | <LOQ | <LOQ | 0.18 | <LOQ | <LOQ | <LOQ |
| **129_H_0072** | 4.85 | 3.19 | 2.21 | 5.08 | 1.96 | 1.59 | 2.73 | <LOQ | 0.04 | 0.12 | <LOQ | <LOQ | <LOQ |
| **130_H_0073** | 22.15 | 20.57 | 0.58 | 3.82 | 0.71 | 0.38 | 0.94 | <LOQ | <LOQ | ND | <LOQ | <LOQ | <LOQ |
| **131_H_0074** | 19.78 | 17.87 | 3.77 | 16.04 | 3.10 | 2.18 | 0.48 | <LOQ | <LOQ | ND | <LOQ | <LOQ | <LOQ |
| **132_H_0075** | ND | ND | 0.56 | ND | 0.45 | 0.40 | 0.84 | <LOQ | <LOQ | 0.42 | <LOQ | <LOQ | 0.14 |
| **133_H_0076** | 30.22 | 9.18 | 0.95 | 34.04 | 0.61 | 0.44 | 3.05 | <LOQ | 0.04 | 0.65 | <LOQ | <LOQ | <LOQ |
| **134_H_0077** | 19.21 | 13.94 | 1.94 | 14.09 | 1.60 | 1.32 | 3.33 | <LOQ | <LOQ | 0.24 | <LOQ | <LOQ | <LOQ |
| **135_H_0078** | 20.55 | 12.40 | 0.80 | 11.20 | 0.47 | 0.36 | 2.14 | <LOQ | <LOQ | 0.05 | <LOQ | <LOQ | <LOQ |
| **136_H_0079** | ND | 0.04 | 0.86 | ND | 0.70 | 0.55 | 0.68 | <LOQ | <LOQ | <LOQ | <LOQ | <LOQ | <LOQ |
| **137_H_0080** | 25.38 | 28.89 | 4.14 | 32.32 | 3.44 | 2.78 | 1.30 | <LOQ | 0.22 | 0.07 | <LOQ | <LOQ | <LOQ |
| **138_H_0081** | ND | ND | 0.41 | ND | 0.35 | 0.25 | 0.38 | <LOQ | <LOQ | <LOQ | <LOQ | <LOQ | <LOQ |
| **139_H_0082** | 7.72 | 0.96 | 1.42 | ND | 1.16 | 1.02 | 2.54 | <LOQ | <LOQ | 0.31 | <LOQ | <LOQ | <LOQ |
| **140_H_0084** | 22.21 | 17.49 | 0.53 | 23.69 | 0.47 | 0.30 | 1.81 | <LOQ | 0.10 | ND | <LOQ | <LOQ | <LOQ |
| **141_H_0085** | 18.37 | 8.89 | 1.52 | 12.34 | 1.19 | 0.88 | 2.56 | <LOQ | <LOQ | 0.05 | <LOQ | <LOQ | <LOQ |
| **142_H_0086** | 9.13 | 6.24 | 2.23 | 3.31 | 1.92 | 1.28 | 2.03 | <LOQ | <LOQ | <LOQ | <LOQ | <LOQ | <LOQ |
| **143_H_0087** | 17.57 | 8.67 | 1.17 | 5.80 | 0.79 | 0.62 | 1.60 | <LOQ | 0.05 | <LOQ | <LOQ | <LOQ | <LOQ |
| **144_H_0088** | 16.91 | 11.19 | 2.78 | 10.16 | 2.04 | 1.28 | 3.53 | <LOQ | 0.05 | 0.05 | <LOQ | <LOQ | <LOQ |
| **145_H_0089** | 17.56 | 9.08 | 0.92 | 10.00 | 0.53 | 0.43 | 0.70 | <LOQ | 0.07 | <LOQ | <LOQ | <LOQ | <LOQ |
| **146_H_0090** | 18.74 | 15.73 | 1.47 | 8.18 | 1.33 | 1.05 | 4.13 | <LOQ | <LOQ | 0.32 | <LOQ | <LOQ | <LOQ |
| **147_H_0091** | 34.66 | 23.81 | 3.17 | 18.15 | 2.55 | 2.05 | 10.00 | <LOQ | <LOQ | 0.31 | <LOQ | <LOQ | <LOQ |
| **148_H_0092** | 39.83 | 33.90 | 1.79 | 38.36 | 1.57 | 1.15 | 5.68 | <LOQ | 0.08 | 0.08 | <LOQ | <LOQ | <LOQ |
| **149_H_0093** | 17.85 | 12.13 | 1.38 | 10.90 | 1.00 | 0.83 | 2.95 | <LOQ | 0.07 | 0.12 | <LOQ | <LOQ | <LOQ |
| **150_H_0094** | 15.28 | 11.17 | 1.55 | 5.43 | 0.66 | 0.53 | 0.24 | <LOQ | 0.06 | <LOQ | <LOQ | <LOQ | <LOQ |
| **151_H_0095** | 25.02 | 23.43 | 2.68 | 14.36 | 1.84 | 1.59 | 3.33 | <LOQ | 0.09 | 0.06 | <LOQ | <LOQ | <LOQ |
| **152_H_0096** | 22.76 | 18.90 | 1.89 | 11.61 | 1.35 | 1.06 | 2.78 | <LOQ | 0.07 | <LOQ | <LOQ | <LOQ | <LOQ |
| **153_H_0097** | 19.05 | 10.75 | 0.48 | 5.74 | 0.31 | 0.18 | 1.40 | <LOQ | <LOQ | <LOQ | <LOQ | <LOQ | <LOQ |
| **154_H_0098** | 30.07 | 16.44 | 1.64 | 12.98 | 1.23 | 0.98 | 2.73 | <LOQ | ND | 0.06 | <LOQ | <LOQ | <LOQ |
| **155_H_0099** | 24.31 | 14.95 | 1.60 | 24.00 | 1.05 | 0.92 | 3.63 | <LOQ | 0.04 | 1.82 | <LOQ | <LOQ | 0.15 |
| **156_H_0100** | 9.67 | 5.76 | 0.74 | 6.34 | 0.51 | 0.37 | 1.41 | <LOQ | <LOQ | 0.40 | <LOQ | <LOQ | 0.05 |
| **157_H_0101** | 10.32 | 10.51 | 0.22 | 6.14 | 0.16 | 0.07 | 0.82 | <LOQ | <LOQ | <LOQ | <LOQ | <LOQ | <LOQ |
| **158_H_0102** | 18.03 | 0.56 | 0.05 | 11.47 | 0.01 | 0.02 | 0.14 | <LOQ | <LOQ | <LOQ | <LOQ | <LOQ | <LOQ |

*ND – Not determined

| **Supplementary Table S7. SCFAs Concentration in Plasma sample (N=157)** | | | | | | | | | | | | | |
| --- | --- | --- | --- | --- | --- | --- | --- | --- | --- | --- | --- | --- | --- |
| **Subject** | **Concentration (µM)** | | | | | | | | | | | | |
|  | **Ace** | **Pro** | **2-Mpro** | **But** | **3-Mbut** | **2-Mbut** | **Pen** | **3-Mpen** | **4-Mpen** | **Hex** | **2-Mhex** | **4-Mhex** | **Hep** |
| **1_G1_C1_003** | 26.64 | 0.41 | 1.95 | 1.25 | 0.97 | 1.06 | 1.18 | 0.75 | 2.97 | 2.30 | 1.05 | 4.01 | 1.45 |
| **2_G1_C1_006** | 36.63 | 0.57 | 1.59 | 1.12 | 0.68 | 1.03 | 1.00 | 0.66 | 2.63 | 1.95 | 0.81 | 3.32 | 1.09 |
| **3_G1_C1_008** | 29.31 | 0.59 | 1.64 | 1.16 | 0.83 | 0.87 | 0.82 | 0.43 | 2.68 | 2.71 | 0.65 | 3.51 | 0.94 |
| **4_G1_C1_009** | 22.65 | 0.65 | 1.66 | 0.91 | 0.69 | 0.85 | 0.82 | 0.46 | 2.63 | 1.74 | 0.63 | 3.25 | 0.81 |
| **5_G1_C1_010** | 54.62 | 0.72 | 1.22 | 0.83 | 0.48 | 0.90 | 0.72 | 0.38 | 2.18 | 1.64 | 0.78 | 2.55 | 0.74 |
| **6_G1_C1_011** | 29.31 | 0.58 | 1.81 | 1.09 | 0.98 | 1.03 | 1.12 | 0.71 | 2.65 | 2.15 | 0.82 | 3.88 | 1.52 |
| **7_G1_C1_012** | 10.66 | 0.37 | 1.56 | 0.98 | 0.71 | 1.17 | 1.06 | 0.64 | 2.77 | 2.34 | 0.86 | 3.46 | 1.23 |
| **8_G1_C1_014** | 37.30 | 0.47 | 1.42 | 0.92 | 0.71 | 0.77 | 0.78 | 0.50 | 2.23 | 1.69 | 0.66 | 3.26 | 0.85 |
| **9_G1_C1_015** | 23.98 | 0.31 | 1.31 | 0.71 | 0.47 | 0.78 | 0.66 | 0.37 | 2.29 | 1.36 | 0.64 | 2.86 | 0.74 |
| **10_G1_C1_018** | 18.65 | 0.52 | 1.42 | 0.73 | 0.60 | 0.97 | 0.70 | 0.40 | 2.27 | 1.57 | 0.59 | 2.78 | 0.68 |
| **11_G1_C2_001** | 11.32 | 0.42 | 1.49 | 0.98 | 0.79 | 0.95 | 0.97 | 0.58 | 2.39 | 1.93 | 1.09 | 3.37 | 1.37 |
| **12_G1_C2_003** | 9.99 | 0.50 | 1.40 | 0.69 | 0.95 | 1.05 | 0.80 | 0.37 | 1.96 | 1.84 | 0.77 | 3.01 | 0.97 |
| **13_G1_C2_004** | 12.66 | 0.56 | 1.16 | 1.02 | 0.64 | 0.65 | 0.74 | 0.31 | 1.84 | 1.56 | 0.67 | 2.30 | 0.75 |
| **14_G1_C2_005** | 16.65 | 0.37 | 1.21 | 0.65 | 0.67 | 0.84 | 0.63 | 0.31 | 2.21 | 1.57 | 0.54 | 2.59 | 0.64 |
| **15_G1_C2_006** | 19.32 | 0.42 | 1.33 | 0.75 | 0.55 | 0.95 | 0.64 | 0.30 | 2.45 | 1.92 | 0.60 | 3.15 | 0.72 |
| **16_G1_C2_007** | 11.32 | 0.28 | 1.26 | 0.63 | 0.29 | 0.55 | 0.60 | 0.26 | 2.33 | 1.63 | 0.57 | 3.12 | 0.63 |
| **17_G1_C2_008** | 27.31 | 0.37 | 1.29 | 0.84 | 0.76 | 0.76 | 0.66 | 0.24 | 2.44 | 2.05 | 0.53 | 3.06 | 0.75 |
| **18_G1_C2_009** | 31.97 | 0.41 | 1.24 | 0.83 | 0.38 | 0.72 | 0.57 | 0.25 | 2.24 | 1.57 | 0.41 | 3.04 | 0.51 |
| **19_G1_C2_010** | 35.30 | 0.41 | 1.59 | 0.61 | 0.35 | 0.65 | 0.52 | 0.21 | 2.98 | 2.03 | 0.39 | 4.05 | 0.56 |
| **20_G1_C2_011** | 26.64 | 0.40 | 1.35 | 0.70 | 0.47 | 0.81 | 0.59 | 0.21 | 2.23 | 1.96 | 0.35 | 3.01 | 0.60 |
| **21_G2_C1_001** | 29.31 | 0.48 | 1.64 | 1.20 | 0.82 | 1.09 | 1.21 | 0.70 | 2.62 | 2.43 | 0.97 | 3.93 | 1.92 |
| **22_G2_C1_003** | 36.63 | 0.45 | 1.60 | 0.84 | 0.74 | 1.26 | 0.87 | 0.48 | 2.52 | 2.11 | 0.78 | 3.34 | 1.05 |
| **23_G2_C1_004** | 17.32 | 0.41 | 1.08 | 0.90 | 0.83 | 0.78 | 0.73 | 0.34 | 2.10 | 1.73 | 0.57 | 2.86 | 0.89 |
| **24_G2_C1_005** | 27.98 | 0.50 | 1.06 | 0.70 | 0.52 | 0.82 | 0.64 | 0.30 | 2.01 | 1.63 | 0.54 | 2.70 | 0.81 |
| **25_G2_C1_006** | 33.97 | 0.43 | 1.09 | 0.73 | 0.95 | 0.74 | 0.58 | 0.26 | 2.23 | 1.59 | 0.54 | 2.70 | 0.59 |
| **26_G2_C1_007** | 41.30 | 0.51 | 1.51 | 1.11 | 0.91 | 0.96 | 1.20 | 0.79 | 2.83 | 3.37 | 1.45 | 3.98 | 2.03 |
| **27_G2_C1_009** | 67.28 | 0.70 | 1.23 | 0.93 | 1.13 | 0.94 | 0.78 | 0.38 | 2.23 | 1.87 | 0.63 | 3.13 | 0.97 |
| **28_G2_C1_010** | 30.64 | 0.55 | 1.25 | 0.73 | 0.70 | 0.85 | 0.70 | 0.37 | 1.72 | 1.52 | 0.83 | 2.50 | 0.72 |
| **29_G2_C1_012** | 58.62 | 0.42 | 1.26 | 0.61 | 0.77 | 0.89 | 0.60 | 0.33 | 2.07 | 1.46 | 0.57 | 2.70 | 0.59 |
| **30_G2_C1_013** | 27.31 | 0.52 | 1.05 | 0.54 | 0.53 | 1.08 | 0.58 | 0.27 | 1.68 | 1.36 | 0.46 | 2.46 | 0.67 |
| **31_G2_C1_014** | 41.30 | 0.69 | 3.33 | 1.69 | 1.32 | 1.59 | 1.78 | 1.13 | 5.39 | 3.93 | 1.59 | 7.66 | 3.14 |
| **32_G2_C1_015** | 27.98 | 0.82 | 2.34 | 1.17 | 0.70 | 0.99 | 1.12 | 0.60 | 3.11 | 2.96 | 0.92 | 5.68 | 1.88 |
| **33_G2_C1_017** | 35.97 | 0.49 | 2.89 | 0.77 | 0.90 | 0.75 | 0.84 | 0.47 | 4.63 | 2.10 | 0.80 | 6.31 | 1.19 |
| **34_G2_C2_001** | 31.31 | 0.53 | 2.72 | 0.92 | 0.90 | 0.91 | 0.85 | 0.45 | 3.40 | 2.27 | 0.67 | 5.87 | 1.08 |
| **35_G2_C2_002** | 140.54 | 0.60 | 2.06 | 0.80 | 0.49 | 0.68 | 0.71 | 0.35 | 2.23 | 1.95 | 0.62 | 4.12 | 0.84 |
| **36_G2_C2_003** | 20.65 | 0.37 | 1.94 | 1.07 | 0.89 | 1.00 | 1.23 | 0.77 | 2.42 | 2.37 | 1.07 | 4.31 | 1.87 |
| **37_G2_C2_005** | 22.65 | 0.52 | 2.07 | 0.95 | 0.62 | 0.84 | 0.87 | 0.48 | 2.76 | 2.08 | 0.68 | 4.43 | 1.12 |
| **38_G2_C2_006** | 17.32 | 0.56 | 1.77 | 0.78 | 0.76 | 0.92 | 0.78 | 0.38 | 1.99 | 1.75 | 0.68 | 3.77 | 0.96 |
| **39_G2_C2_007** | 15.99 | 0.52 | 1.90 | 0.61 | 0.51 | 0.81 | 0.71 | 0.35 | 2.16 | 1.59 | 0.51 | 4.03 | 0.71 |
| **40_G2_C2_008** | 37.30 | 0.44 | 1.92 | 0.64 | 0.65 | 0.56 | 0.66 | 0.29 | 2.79 | 1.96 | 0.48 | 4.16 | 0.91 |
| **41_G3_C1_001** | 15.99 | 0.60 | 0.50 | 1.15 | 0.99 | 1.23 | 1.29 | 0.79 | 1.80 | 2.29 | 1.18 | 2.92 | 1.93 |
| **42_G3_C1_002** | 11.32 | 0.59 | 0.95 | 0.76 | 0.71 | 0.89 | 0.82 | 0.47 | 1.57 | 1.73 | 0.73 | 2.33 | 1.12 |
| **43_G3_C1_003** | 27.31 | 0.74 | 1.59 | 0.86 | 0.75 | 0.83 | 0.75 | 0.36 | 2.06 | 1.68 | 0.74 | 3.29 | 0.87 |
| **44_G3_C1_004** | 20.65 | 0.43 | 0.79 | 0.67 | 0.58 | 0.78 | 0.59 | 0.31 | 1.10 | 0.93 | 0.54 | 1.71 | 0.51 |
| **45_G3_C1_005** | 11.32 | 0.47 | 0.87 | 0.72 | 0.57 | 0.68 | 0.61 | 0.28 | 1.24 | 1.14 | 0.52 | 2.03 | 0.61 |
| **46_G3_C1_010** | 33.97 | 0.44 | 1.64 | 0.62 | 0.51 | 0.87 | 0.57 | 0.25 | 2.08 | 1.45 | 0.60 | 3.43 | 0.57 |
| **47_G3_C1_011** | 26.64 | 0.40 | 1.52 | 0.48 | 0.54 | 0.62 | 0.56 | 0.24 | 2.01 | 1.49 | 0.44 | 3.05 | 0.48 |
| **48_G3_C1_012** | 25.31 | 0.54 | 1.66 | 0.67 | 0.43 | 0.56 | 0.53 | 0.19 | 2.44 | 1.52 | 0.43 | 3.62 | 0.48 |
| **49_G3_C1_013** | 36.63 | 0.47 | 0.76 | 0.54 | 0.68 | 0.70 | 0.49 | 0.19 | 1.14 | 1.16 | 0.44 | 1.66 | 0.43 |
| **50_G3_C1_014** | 9.99 | 0.35 | 1.51 | 0.43 | 0.46 | 0.53 | 0.52 | 0.18 | 2.16 | 1.47 | 0.46 | 3.31 | 0.41 |
| **51_G3_C1_015** | 46.63 | 0.52 | 1.89 | 1.35 | 1.06 | 1.22 | 1.28 | 0.74 | 2.67 | 2.49 | 1.04 | 4.50 | 1.87 |
| **52_G3_C1_016** | 46.63 | 0.57 | 1.13 | 0.81 | 0.68 | 0.84 | 0.90 | 0.48 | 1.71 | 1.68 | 0.85 | 2.63 | 1.21 |
| **53_G3_C1_017** | 29.97 | 0.53 | 1.05 | 0.74 | 0.53 | 0.84 | 0.67 | 0.30 | 1.75 | 2.58 | 0.66 | 2.62 | 0.72 |
| **54_G3_C2_001** | 21.31 | 0.73 | 0.97 | 0.88 | 0.52 | 0.64 | 0.65 | 0.30 | 1.51 | 1.29 | 0.49 | 2.22 | 0.66 |
| **55_G3_C2_002** | 43.30 | 0.57 | 1.45 | 0.55 | 0.77 | 0.71 | 0.59 | 0.23 | 2.00 | 1.29 | 0.37 | 3.39 | 0.68 |
| **56_G3_C2_003** | 39.97 | 0.65 | 1.81 | 1.19 | 1.07 | 0.98 | 1.26 | 0.78 | 3.08 | 2.60 | 1.00 | 4.75 | 1.99 |
| **57_G3_C2_004** | 39.97 | 0.43 | 1.83 | 0.84 | 0.82 | 1.01 | 0.82 | 0.45 | 2.54 | 2.03 | 0.69 | 4.34 | 1.05 |
| **58_G3_C2_005** | 40.63 | 0.55 | 1.39 | 0.67 | 0.59 | 0.75 | 0.71 | 0.34 | 1.91 | 1.82 | 0.61 | 3.03 | 0.93 |
| **59_G3_C2_006** | 27.98 | 0.51 | 1.20 | 0.73 | 0.68 | 0.75 | 0.59 | 0.23 | 1.48 | 1.50 | 0.50 | 2.41 | 0.63 |
| **60_G3_C2_007** | 43.96 | 0.44 | 0.92 | 0.71 | 0.95 | 0.80 | 0.51 | 0.23 | 1.01 | 1.32 | 0.42 | 1.78 | 0.54 |
| **61_H_0001** | 108.18 | 4.42 | <LOQ | <LOQ | <LOQ | <LOQ | <LOQ | <LOQ | <LOQ | <LOQ | <LOQ | <LOQ | <LOQ |
| **62_H_0002** | 81.51 | 4.22 | <LOQ | <LOQ | <LOQ | <LOQ | <LOQ | <LOQ | <LOQ | <LOQ | <LOQ | <LOQ | <LOQ |
| **63_H_0003** | 204.44 | 3.89 | <LOQ | <LOQ | <LOQ | <LOQ | <LOQ | <LOQ | <LOQ | <LOQ | <LOQ | <LOQ | <LOQ |
| **64_H_0004** | 265.98 | 4.57 | <LOQ | 0.12 | <LOQ | <LOQ | <LOQ | <LOQ | <LOQ | 0.22 | <LOQ | <LOQ | <LOQ |
| **65_H_0005** | 295.20 | 3.58 | <LOQ | <LOQ | <LOQ | <LOQ | <LOQ | <LOQ | <LOQ | <LOQ | <LOQ | <LOQ | <LOQ |
| **66_H_0006** | 261.21 | 20.61 | <LOQ | 5.71 | <LOQ | <LOQ | <LOQ | <LOQ | <LOQ | 0.56 | <LOQ | <LOQ | <LOQ |
| **67_H_0007** | ND | ND | <LOQ | <LOQ | <LOQ | <LOQ | <LOQ | <LOQ | <LOQ | <LOQ | <LOQ | <LOQ | <LOQ |
| **68_H_0008** | ND | ND | <LOQ | <LOQ | <LOQ | <LOQ | <LOQ | <LOQ | <LOQ | <LOQ | <LOQ | <LOQ | <LOQ |
| **69_H_0009** | 231.81 | 3.92 | 0.35 | <LOQ | <LOQ | <LOQ | <LOQ | <LOQ | <LOQ | <LOQ | <LOQ | <LOQ | <LOQ |
| **70_H_0010** | 232.72 | 3.68 | <LOQ | <LOQ | <LOQ | <LOQ | <LOQ | <LOQ | <LOQ | <LOQ | <LOQ | <LOQ | <LOQ |
| **71_H_0011** | 247.65 | 3.32 | <LOQ | <LOQ | <LOQ | <LOQ | <LOQ | <LOQ | <LOQ | <LOQ | <LOQ | <LOQ | <LOQ |
| **72_H_0012** | 109.33 | 4.49 | <LOQ | <LOQ | <LOQ | <LOQ | <LOQ | <LOQ | <LOQ | <LOQ | <LOQ | <LOQ | <LOQ |
| **73_H_0013** | 76.95 | 4.37 | <LOQ | <LOQ | <LOQ | <LOQ | <LOQ | <LOQ | <LOQ | 0.01 | <LOQ | <LOQ | <LOQ |
| **74_H_0014** | 147.32 | 9.93 | <LOQ | 1.63 | <LOQ | <LOQ | <LOQ | <LOQ | <LOQ | <LOQ | <LOQ | <LOQ | <LOQ |
| **75_H_0015** | 262.85 | 3.75 | <LOQ | <LOQ | <LOQ | <LOQ | <LOQ | <LOQ | <LOQ | <LOQ | <LOQ | <LOQ | <LOQ |
| **76_H_0016** | 62.83 | 3.31 | <LOQ | <LOQ | <LOQ | <LOQ | <LOQ | <LOQ | <LOQ | <LOQ | <LOQ | <LOQ | <LOQ |
| **77_H_0017** | 217.28 | 4.31 | <LOQ | <LOQ | <LOQ | <LOQ | <LOQ | <LOQ | <LOQ | <LOQ | <LOQ | <LOQ | <LOQ |
| **78_H_0018** | 84.60 | 3.85 | <LOQ | <LOQ | <LOQ | <LOQ | <LOQ | <LOQ | <LOQ | <LOQ | <LOQ | <LOQ | <LOQ |
| **80_H_0021** | 39.27 | 3.26 | <LOQ | <LOQ | <LOQ | <LOQ | <LOQ | <LOQ | <LOQ | <LOQ | <LOQ | <LOQ | <LOQ |
| **81_H_0022** | 40.29 | 4.53 | <LOQ | <LOQ | <LOQ | <LOQ | <LOQ | <LOQ | <LOQ | <LOQ | <LOQ | <LOQ | <LOQ |
| **82_H_0023** | 144.64 | 3.23 | <LOQ | <LOQ | <LOQ | <LOQ | <LOQ | <LOQ | <LOQ | <LOQ | <LOQ | <LOQ | <LOQ |
| **83_H_0024** | 108.99 | 3.32 | <LOQ | <LOQ | <LOQ | <LOQ | <LOQ | <LOQ | <LOQ | <LOQ | <LOQ | <LOQ | <LOQ |
| **84_H_0025** | 91.37 | 3.69 | <LOQ | <LOQ | <LOQ | <LOQ | <LOQ | <LOQ | <LOQ | <LOQ | <LOQ | <LOQ | <LOQ |
| **85_H_0026** | 52.74 | ND | <LOQ | <LOQ | <LOQ | <LOQ | <LOQ | <LOQ | <LOQ | <LOQ | <LOQ | <LOQ | <LOQ |
| **86_H_0027** | 43.40 | 3.03 | <LOQ | <LOQ | <LOQ | <LOQ | <LOQ | <LOQ | <LOQ | <LOQ | <LOQ | <LOQ | <LOQ |
| **87_H_0028** | 48.23 | 3.71 | <LOQ | <LOQ | <LOQ | <LOQ | <LOQ | <LOQ | <LOQ | <LOQ | <LOQ | <LOQ | <LOQ |
| **88_H_0029** | 41.66 | 3.08 | <LOQ | <LOQ | <LOQ | <LOQ | <LOQ | <LOQ | <LOQ | <LOQ | <LOQ | <LOQ | <LOQ |
| **89_H_0030** | 43.72 | 3.73 | 0.59 | <LOQ | <LOQ | <LOQ | <LOQ | <LOQ | <LOQ | <LOQ | <LOQ | <LOQ | <LOQ |
| **90_H_0031** | 56.03 | 5.27 | 0.69 | <LOQ | <LOQ | <LOQ | <LOQ | <LOQ | <LOQ | <LOQ | <LOQ | <LOQ | <LOQ |
| **91_H_0032** | 58.19 | 4.61 | 0.47 | <LOQ | <LOQ | <LOQ | <LOQ | <LOQ | <LOQ | <LOQ | <LOQ | <LOQ | <LOQ |
| **92_H_0033** | 44.25 | 3.65 | <LOQ | <LOQ | <LOQ | <LOQ | <LOQ | <LOQ | <LOQ | <LOQ | <LOQ | <LOQ | <LOQ |
| **93_H_0034** | 144.35 | 6.29 | 2.58 | <LOQ | <LOQ | <LOQ | <LOQ | <LOQ | <LOQ | <LOQ | <LOQ | <LOQ | <LOQ |
| **94_H_0035** | 226.59 | 3.85 | <LOQ | <LOQ | <LOQ | <LOQ | <LOQ | <LOQ | <LOQ | <LOQ | <LOQ | <LOQ | <LOQ |
| **95_H_0036** | 59.88 | 2.93 | 0.67 | <LOQ | <LOQ | <LOQ | <LOQ | <LOQ | <LOQ | <LOQ | <LOQ | <LOQ | <LOQ |
| **96_H_0037** | 135.55 | 3.68 | 0.51 | <LOQ | <LOQ | <LOQ | <LOQ | <LOQ | <LOQ | <LOQ | <LOQ | <LOQ | <LOQ |
| **97_H_0038** | 59.92 | 3.39 | 1.13 | <LOQ | <LOQ | <LOQ | <LOQ | <LOQ | <LOQ | <LOQ | <LOQ | <LOQ | <LOQ |
| **98_H_0039** | 50.48 | 3.52 | 0.26 | <LOQ | <LOQ | <LOQ | <LOQ | <LOQ | <LOQ | <LOQ | <LOQ | <LOQ | <LOQ |
| **99_H_0040** | 129.85 | 4.04 | <LOQ | <LOQ | <LOQ | <LOQ | <LOQ | <LOQ | <LOQ | <LOQ | <LOQ | <LOQ | <LOQ |
| **100_H_0041** | 179.88 | 4.41 | <LOQ | <LOQ | <LOQ | <LOQ | <LOQ | <LOQ | <LOQ | <LOQ | <LOQ | <LOQ | <LOQ |
| **101_H_0042** | 49.87 | 3.98 | <LOQ | <LOQ | <LOQ | <LOQ | <LOQ | <LOQ | <LOQ | <LOQ | <LOQ | <LOQ | <LOQ |
| **102_H_0043** | 116.25 | 3.91 | <LOQ | <LOQ | <LOQ | <LOQ | <LOQ | <LOQ | <LOQ | <LOQ | <LOQ | <LOQ | <LOQ |
| **103_H_0044** | 48.48 | 3.79 | <LOQ | <LOQ | <LOQ | <LOQ | <LOQ | <LOQ | <LOQ | <LOQ | <LOQ | <LOQ | <LOQ |
| **104_H_0045** | 59.34 | 3.93 | <LOQ | <LOQ | <LOQ | <LOQ | <LOQ | <LOQ | <LOQ | <LOQ | <LOQ | <LOQ | <LOQ |
| **105_H_0046** | 43.48 | 3.88 | <LOQ | <LOQ | <LOQ | <LOQ | <LOQ | <LOQ | <LOQ | <LOQ | <LOQ | <LOQ | <LOQ |
| **106_H_0047** | 137.25 | 3.32 | <LOQ | <LOQ | <LOQ | <LOQ | <LOQ | <LOQ | <LOQ | <LOQ | <LOQ | <LOQ | <LOQ |
| **107_H_0048** | 134.39 | 3.57 | <LOQ | <LOQ | <LOQ | <LOQ | <LOQ | <LOQ | <LOQ | <LOQ | <LOQ | <LOQ | <LOQ |
| **108_H_0049** | 54.47 | 3.42 | <LOQ | <LOQ | <LOQ | <LOQ | <LOQ | <LOQ | <LOQ | <LOQ | <LOQ | <LOQ | <LOQ |
| **109_H_0050** | 42.44 | 3.61 | 0.07 | <LOQ | <LOQ | <LOQ | <LOQ | <LOQ | <LOQ | <LOQ | <LOQ | <LOQ | <LOQ |
| **110_H_0051** | 31.23 | 2.96 | 0.17 | <LOQ | <LOQ | <LOQ | <LOQ | <LOQ | <LOQ | <LOQ | <LOQ | <LOQ | <LOQ |
| **111_H_0052** | 47.25 | 3.41 | 0.08 | <LOQ | <LOQ | <LOQ | <LOQ | <LOQ | <LOQ | <LOQ | <LOQ | <LOQ | <LOQ |
| **112_H_0053** | 41.25 | 3.11 | 0.14 | <LOQ | <LOQ | <LOQ | <LOQ | <LOQ | <LOQ | <LOQ | <LOQ | <LOQ | <LOQ |
| **113_H_0054** | 34.22 | 3.68 | 0.30 | <LOQ | <LOQ | <LOQ | <LOQ | <LOQ | <LOQ | <LOQ | <LOQ | <LOQ | <LOQ |
| **114_H_0055** | 55.12 | 3.65 | 0.26 | <LOQ | <LOQ | <LOQ | <LOQ | <LOQ | <LOQ | <LOQ | <LOQ | <LOQ | <LOQ |
| **115_H_0056** | 83.28 | 3.75 | <LOQ | <LOQ | <LOQ | <LOQ | <LOQ | <LOQ | <LOQ | <LOQ | <LOQ | <LOQ | <LOQ |
| **116_H_0057** | 39.00 | 3.59 | <LOQ | <LOQ | <LOQ | <LOQ | <LOQ | <LOQ | <LOQ | <LOQ | <LOQ | <LOQ | <LOQ |
| **117_H_0058** | 59.90 | ND | 0.42 | <LOQ | <LOQ | <LOQ | <LOQ | <LOQ | <LOQ | <LOQ | <LOQ | <LOQ | <LOQ |
| **118_H_0059** | 61.41 | 4.18 | 0.93 | <LOQ | <LOQ | <LOQ | <LOQ | <LOQ | <LOQ | <LOQ | <LOQ | <LOQ | <LOQ |
| **119_H_0060** | 112.82 | 3.84 | <LOQ | <LOQ | <LOQ | <LOQ | <LOQ | <LOQ | <LOQ | <LOQ | <LOQ | <LOQ | <LOQ |
| **120_H_0061** | 70.06 | 4.08 | 0.08 | <LOQ | <LOQ | <LOQ | <LOQ | <LOQ | <LOQ | <LOQ | <LOQ | <LOQ | <LOQ |
| **121_H_0062** | 49.17 | 3.88 | <LOQ | <LOQ | <LOQ | <LOQ | <LOQ | <LOQ | <LOQ | <LOQ | <LOQ | <LOQ | <LOQ |
| **122_H_0064** | 52.18 | 3.51 | <LOQ | <LOQ | <LOQ | <LOQ | <LOQ | <LOQ | <LOQ | <LOQ | <LOQ | <LOQ | <LOQ |
| **123_H_0065** | 136.06 | 5.55 | 0.75 | <LOQ | <LOQ | <LOQ | <LOQ | <LOQ | <LOQ | <LOQ | <LOQ | <LOQ | <LOQ |
| **124_H_0067** | 121.31 | ND | <LOQ | <LOQ | <LOQ | <LOQ | <LOQ | <LOQ | <LOQ | <LOQ | <LOQ | <LOQ | <LOQ |
| **125_H_0068** | 122.46 | 4.71 | <LOQ | <LOQ | <LOQ | <LOQ | <LOQ | <LOQ | <LOQ | <LOQ | <LOQ | <LOQ | <LOQ |
| **126_H_0069** | 70.46 | 4.26 | <LOQ | <LOQ | <LOQ | <LOQ | <LOQ | <LOQ | <LOQ | <LOQ | <LOQ | <LOQ | <LOQ |
| **127_H_0070** | 112.67 | 4.30 | 1.21 | <LOQ | <LOQ | <LOQ | <LOQ | <LOQ | <LOQ | <LOQ | <LOQ | <LOQ | <LOQ |
| **128_H_0071** | 115.53 | 4.00 | <LOQ | <LOQ | <LOQ | <LOQ | <LOQ | <LOQ | <LOQ | <LOQ | <LOQ | <LOQ | <LOQ |
| **129_H_0072** | 71.14 | 3.96 | <LOQ | <LOQ | <LOQ | <LOQ | <LOQ | <LOQ | <LOQ | <LOQ | <LOQ | <LOQ | <LOQ |
| **130_H_0073** | 63.12 | 4.85 | <LOQ | <LOQ | <LOQ | <LOQ | <LOQ | <LOQ | <LOQ | <LOQ | <LOQ | <LOQ | <LOQ |
| **131_H_0074** | 44.33 | 4.22 | <LOQ | <LOQ | <LOQ | <LOQ | <LOQ | <LOQ | <LOQ | <LOQ | <LOQ | <LOQ | <LOQ |
| **132_H_0075** | 120.23 | 3.56 | <LOQ | <LOQ | <LOQ | <LOQ | <LOQ | <LOQ | <LOQ | <LOQ | <LOQ | <LOQ | <LOQ |
| **133_H_0076** | 131.36 | 5.46 | <LOQ | <LOQ | <LOQ | <LOQ | <LOQ | <LOQ | <LOQ | <LOQ | <LOQ | <LOQ | <LOQ |
| **134_H_0077** | 54.69 | 5.32 | <LOQ | <LOQ | <LOQ | <LOQ | <LOQ | <LOQ | <LOQ | <LOQ | <LOQ | <LOQ | <LOQ |
| **135_H_0078** | 143.69 | 4.81 | <LOQ | <LOQ | <LOQ | <LOQ | <LOQ | <LOQ | <LOQ | <LOQ | <LOQ | <LOQ | <LOQ |
| **136_H_0079** | 129.86 | 4.61 | <LOQ | <LOQ | <LOQ | <LOQ | <LOQ | <LOQ | <LOQ | <LOQ | <LOQ | <LOQ | <LOQ |
| **137_H_0080** | 55.16 | 5.11 | <LOQ | <LOQ | <LOQ | <LOQ | <LOQ | <LOQ | <LOQ | <LOQ | <LOQ | <LOQ | <LOQ |
| **138_H_0081** | 121.31 | 4.84 | 1.33 | <LOQ | <LOQ | <LOQ | <LOQ | <LOQ | <LOQ | <LOQ | <LOQ | <LOQ | <LOQ |
| **139_H_0082** | 51.08 | 4.81 | 1.50 | <LOQ | <LOQ | <LOQ | <LOQ | <LOQ | <LOQ | <LOQ | <LOQ | <LOQ | <LOQ |
| **140_H_0084** | 47.74 | 4.36 | <LOQ | <LOQ | <LOQ | <LOQ | <LOQ | <LOQ | <LOQ | 0.12 | <LOQ | <LOQ | <LOQ |
| **141_H_0085** | 174.32 | ND | <LOQ | <LOQ | <LOQ | <LOQ | <LOQ | <LOQ | <LOQ | <LOQ | <LOQ | <LOQ | <LOQ |
| **142_H_0086** | 171.98 | 3.83 | <LOQ | <LOQ | <LOQ | <LOQ | <LOQ | <LOQ | <LOQ | <LOQ | <LOQ | <LOQ | <LOQ |
| **143_H_0087** | 64.54 | 5.07 | 0.31 | <LOQ | <LOQ | <LOQ | <LOQ | <LOQ | <LOQ | <LOQ | <LOQ | <LOQ | <LOQ |
| **144_H_0088** | 260.39 | 24.67 | 0.08 | 5.44 | <LOQ | <LOQ | <LOQ | <LOQ | <LOQ | <LOQ | <LOQ | <LOQ | <LOQ |
| **145_H_0089** | 253.06 | 8.27 | <LOQ | 0.44 | <LOQ | <LOQ | <LOQ | <LOQ | <LOQ | <LOQ | <LOQ | <LOQ | <LOQ |
| **146_H_0090** | 216.95 | 4.99 | <LOQ | <LOQ | <LOQ | <LOQ | <LOQ | <LOQ | <LOQ | <LOQ | <LOQ | <LOQ | <LOQ |
| **147_H_0091** | 275.14 | 4.21 | 0.03 | <LOQ | <LOQ | <LOQ | <LOQ | <LOQ | <LOQ | <LOQ | <LOQ | <LOQ | <LOQ |
| **148_H_0092** | 155.06 | 6.78 | <LOQ | 0.16 | <LOQ | <LOQ | <LOQ | <LOQ | <LOQ | <LOQ | <LOQ | <LOQ | <LOQ |
| **149_H_0093** | 155.07 | 4.82 | <LOQ | <LOQ | <LOQ | <LOQ | <LOQ | <LOQ | <LOQ | <LOQ | <LOQ | <LOQ | <LOQ |
| **150_H_0094** | 160.97 | 4.48 | 0.21 | <LOQ | <LOQ | <LOQ | <LOQ | <LOQ | <LOQ | <LOQ | <LOQ | <LOQ | <LOQ |
| **151_H_0095** | 132.08 | 4.14 | 0.04 | <LOQ | <LOQ | <LOQ | <LOQ | <LOQ | <LOQ | <LOQ | <LOQ | <LOQ | <LOQ |
| **152_H_0096** | 190.79 | 3.70 | <LOQ | <LOQ | <LOQ | <LOQ | <LOQ | <LOQ | <LOQ | <LOQ | <LOQ | <LOQ | <LOQ |
| **153_H_0097** | 182.84 | 3.76 | 0.15 | <LOQ | <LOQ | <LOQ | <LOQ | <LOQ | <LOQ | <LOQ | <LOQ | <LOQ | <LOQ |
| **154_H_0098** | 179.04 | 4.20 | <LOQ | <LOQ | <LOQ | <LOQ | <LOQ | <LOQ | <LOQ | <LOQ | <LOQ | <LOQ | <LOQ |
| **155_H_0099** | 195.22 | 4.55 | <LOQ | <LOQ | <LOQ | <LOQ | <LOQ | <LOQ | <LOQ | <LOQ | <LOQ | <LOQ | <LOQ |
| **156_H_0100** | 222.82 | 3.97 | <LOQ | <LOQ | <LOQ | <LOQ | <LOQ | <LOQ | <LOQ | <LOQ | <LOQ | <LOQ | <LOQ |
| **157_H_0101** | 186.61 | 3.85 | <LOQ | <LOQ | <LOQ | <LOQ | <LOQ | <LOQ | <LOQ | <LOQ | <LOQ | <LOQ | <LOQ |
| **158_H_0102** | 140.56 | 4.31 | <LOQ | <LOQ | <LOQ | <LOQ | <LOQ | <LOQ | <LOQ | 0.39 | <LOQ | <LOQ | <LOQ |

*ND – Not determined

| **Table S8. Comparison of major SCFAs in feces and plasma samples among Thai, UK, and Malaysian population** | | | | |
| --- | --- | --- | --- | --- |
| **Fecal SCFAs** | **Concentration (µmol/g; Mean ± SD)** | | |  |
| **Major SCFAs** | **Thai population  (N=157)** | **UK population  (N=3)** | **Malaysian population  (N=50)** |  |
| **Ace** | 14.91 ± 10.44 | 97.85 ± 31.28 | 209.7 ± 14 |  |
| **Pro** | 10.70 ± 7.69 | 26.02 ± 14.47 | 93.3 ± 5.3 |  |
| **But** | 9.23 ± 7.54 | 24.69 ± 9.39 | 176 ± 16.0 |  |

| **Plasma SCFAs** | **Concentration (µM; Mean ± SD)** | |
| --- | --- | --- |
| **Major SCFAs** | **Thai population  (N=157)** | **UK population  (N=3)** |
| **Ace** | 83.84 ± 71.04 | 66.92 ± 7.13 |
| **Pro** | 2.93 ± 3.02 | 1.38 ± 0.19 |
| **But** | 0.40 ± 0.74 | 0.76 ± 0.25 |
