# Supplementary figures and images for: Quantifying fecal and plasma short-chain fatty acids in healthy Thai individuals
Source: Comput Struct Biotechnol J. 2024 May 8;23:2163–72. doi: 10.1016/j.csbj.2024.05.007 (PMC11141283; doi:10.1016/j.csbj.2024.05.007)

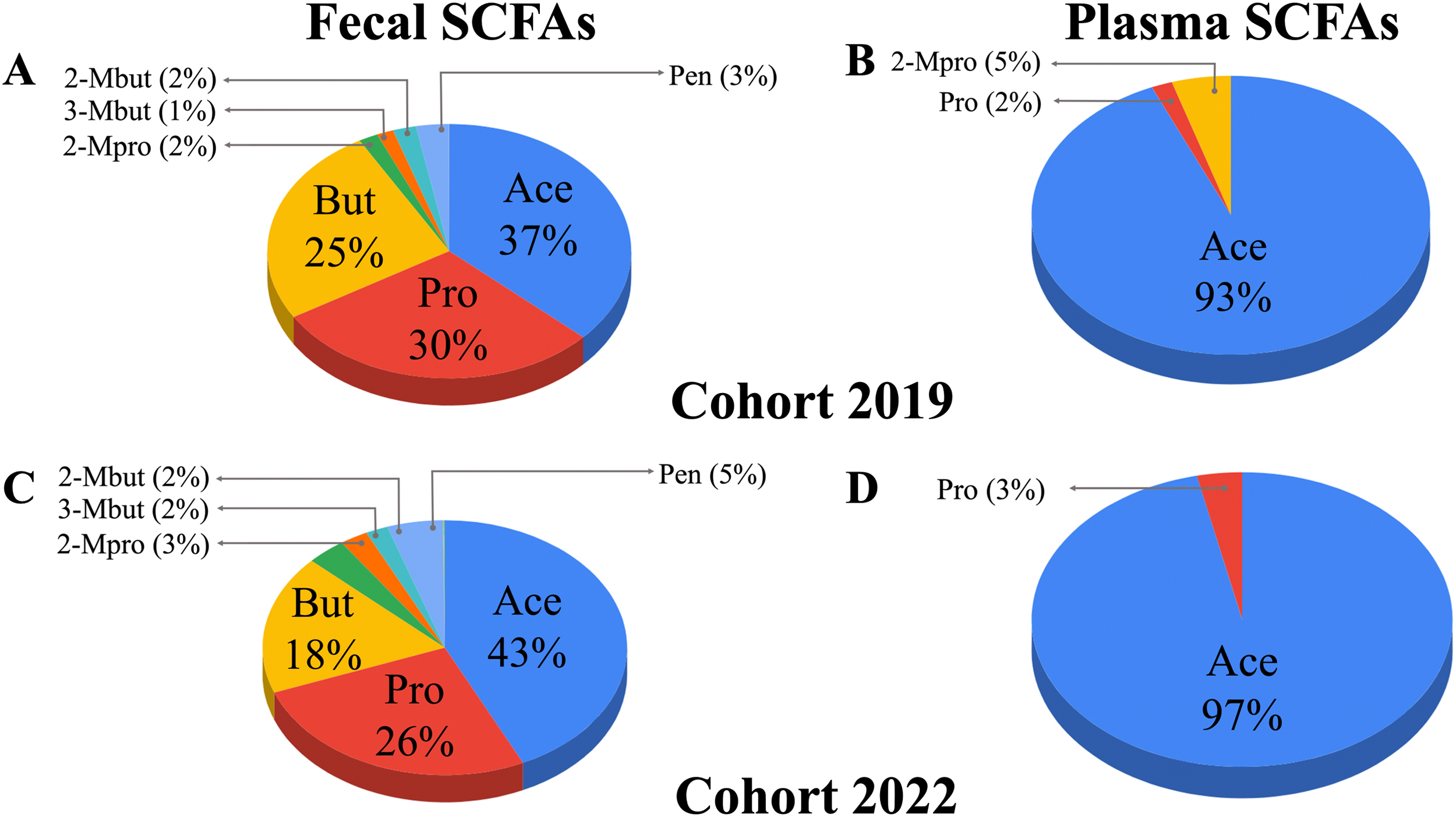

Supplement: Supplementary file 3 — Supplementary material [file mmc3.jpg]

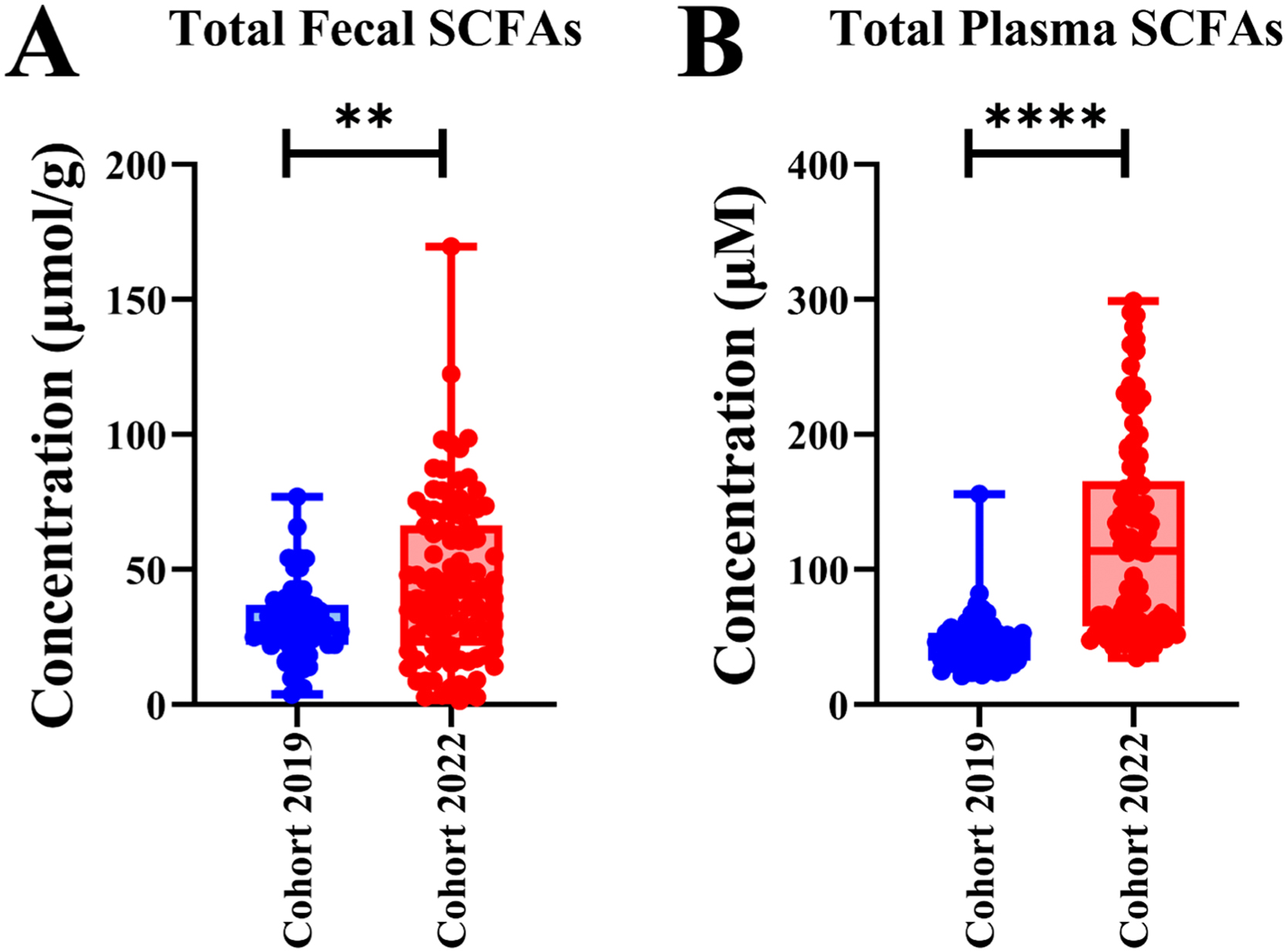

Supplement: Supplementary file 4 — Supplementary material [file mmc4.jpg]

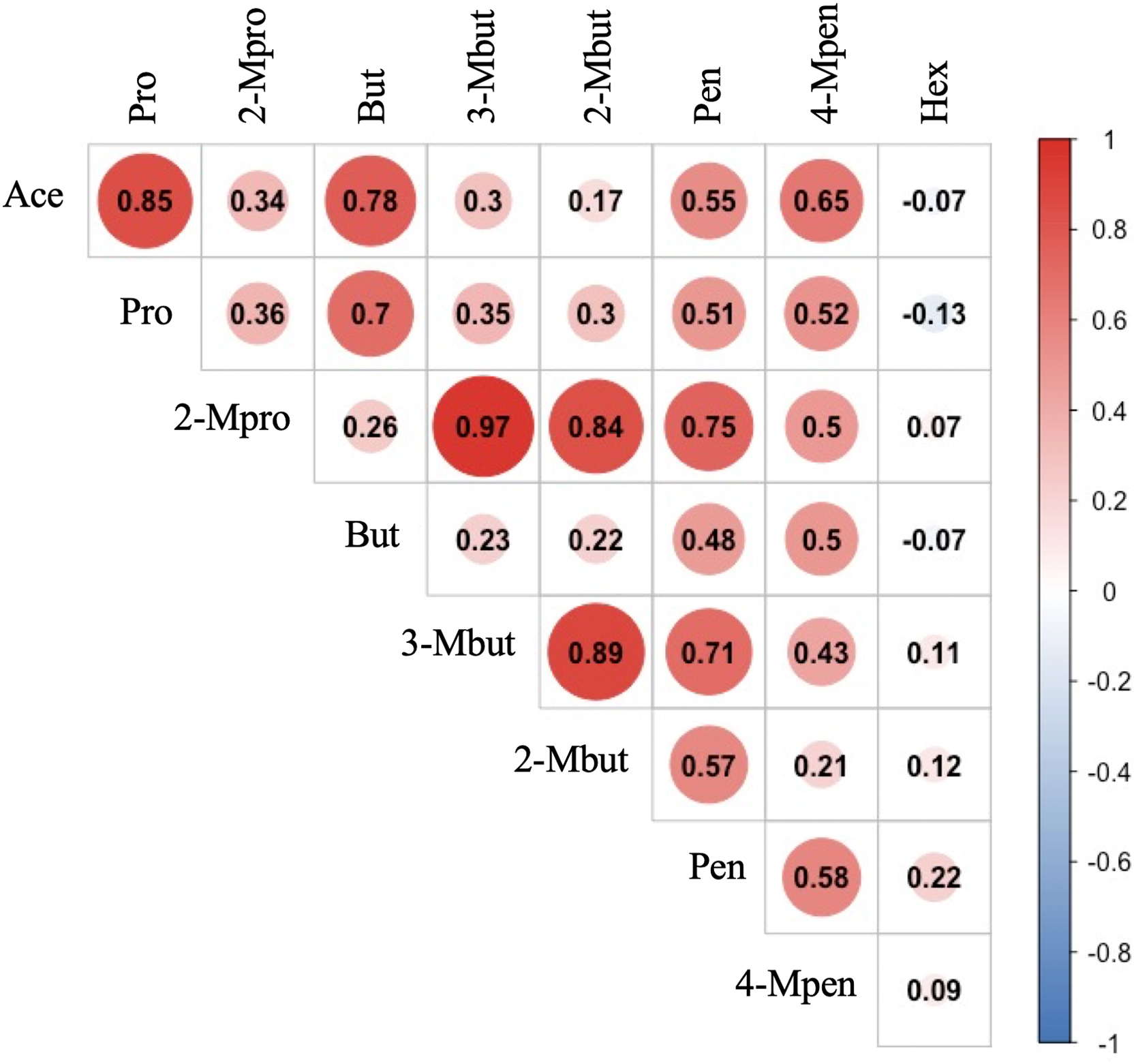

Supplement: Supplementary file 5 — Supplementary material [file mmc5.jpg]

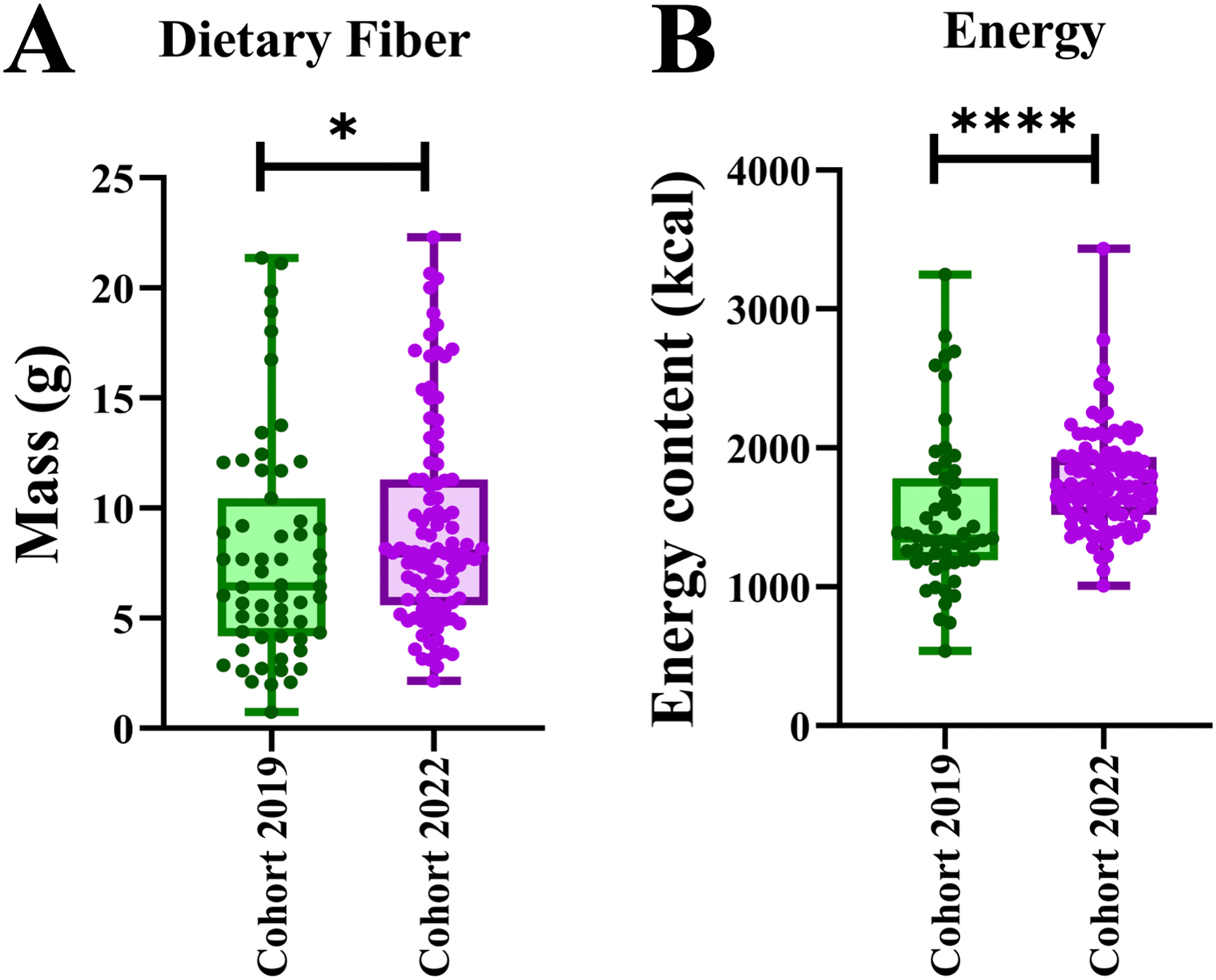

Supplement: Supplementary file 6 — Supplementary material [file mmc6.jpg]

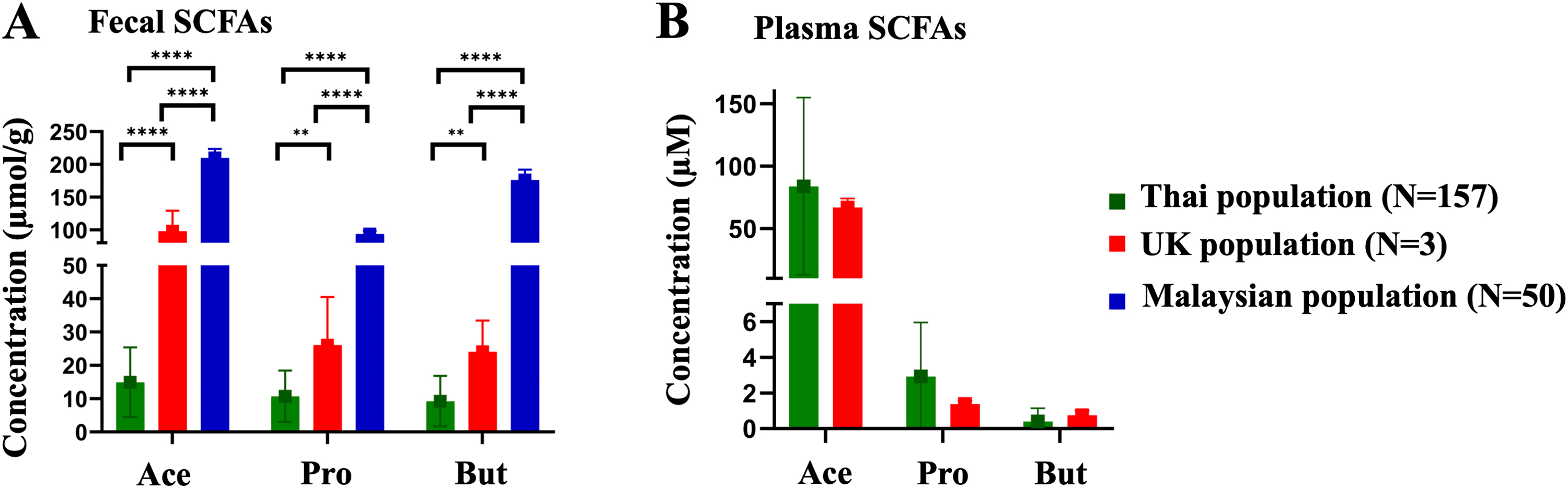

Supplement: Supplementary file 7 — Supplementary material [file mmc7.jpg]
